# Antimicrobial activity, in vitro anticancer effect (MCF-7 breast cancer cell line), antiangiogenic and immunomodulatory potentials of *Populus nigra* L. buds extract

**DOI:** 10.1186/s12906-022-03526-z

**Published:** 2022-03-16

**Authors:** Brigitta Kis, Ioana Zinuca Pavel, Stefana Avram, Elena Alina Moaca, Martina Herrero San Juan, Anja Schwiebs, Heinfried H. Radeke, Delia Muntean, Zorita Diaconeasa, Daliana Minda, Camelia Oprean, Florina Bojin, Cristina Adriana Dehelean, Codruta Soica, Corina Danciu

**Affiliations:** 1 Department of Pharmacognosy, “Victor Babeș” University of Medicine and Pharmacy Timișoara, Romania, Eftimie Murgu Sq. no. 2, 300041 Timișoara, Romania; 2 Research Center for Pharmaco-Toxicological Evaluation, “Victor Babeș” University of Medicine and Pharmacy Timișoara, Romania, Eftimie Murgu Sq. no. 2, 300041 Timișoara, Romania; 3 Department of Toxicology, “Victor Babeș” University of Medicine and Pharmacy Timișoara, Romania, Eftimie Murgu Sq. no. 2, 300041 Timișoara, Romania; 4 Pharmazentrum frankfurt/ZAFES, Institute of General Pharmacology and Toxicology, Hospital of the Goethe University, 60590 Frankfurt/Main, Germany; 5 Department of Microbiology, “Victor Babeș” University of Medicine and Pharmacy Timișoara, Romania, Eftimie Murgu Sq. no. 2, 300041 Timișoara, Romania; 6 Department of Food Science and Technology, Faculty of Food Science and Technology, University of Agricultural Science and Veterinary Medicine, Calea Manastur, 3–5, 400372 Cluj-Napoca, Romania; 7 Department of Pharmacy I, Drug Analysis, “Victor Babeș” University of Medicine and Pharmacy Timișoara, Romania, Eftimie Murgu Sq. no. 2, 300041 Timișoara, Romania; 8 “Pius Brinzeu” Timişoara County Emergency Clinical Hospital, Oncogen Institute, 156 Liviu Rebreanu, 300723 Timişoara, Romania; 9 Advanced Instrumental Screening Center, Faculty of Pharmacy, “Victor Babeș” University of Medicine and Pharmacy Timișoara, Romania, Eftimie Murgu Sq. no. 2, 300041 Timișoara, Romania; 10 Department of Functional Sciences, Faculty of Medicine, “Victor Babeș” University of Medicine and Pharmacy Timișoara, Romania, Eftimie Murgu Sq. no. 2, 300041 Timișoara, Romania; 11 Department of Pharmaceutical Chemistry, “Victor Babeș” University of Medicine and Pharmacy Timișoara, Romania, Eftimie Murgu Sq. no. 2, 300041 Timișoara, Romania

**Keywords:** *Populus nigra* L. buds, Antibacterial, MCF-7 breast cancer cell line, Chorioallantoic membrane, Dendritic cells

## Abstract

**Purpose:**

The aim of this study was to evaluate the antioxidant potential, antimicrobial activity, the in vitro anticancer effect (tested on MCF-7 breast cancer cell line), as well as the antiangiogenic and immunomodulatory potential of *Populus nigra* L. bud (Pg) extract collected from the western part of Romania.

**Results:**

*Populus nigra* L. bud extract presents an important antioxidant activity, due to the rich phytochemical composition. Regarding the biological activity, results have shown that poplar bud extract presents a significant inhibitory activity against Gram-positive bacteria and a dose-dependent decrease of MCF-7 tumor cell viability with an IC_50_ of 66.26 μg/mL, while not affecting healthy cells. Phenomena of early apoptotic events at the maximum concentration tested (150 μg/mL) were detected by Annexin V-PI double staining. The extract induced G0/G1 phase cell cycle arrest. In addition, Pg extract showed antiangiogenic potential on the chorioallantoic membrane. Also, at the highest concentration (150 μg/mL), good tolerability and no signs of toxicity upon vascular plexus were observed. Moreover, in low concentrations, the Pg extract had immunomodulatory activity on primary human dendritic cells by upregulating IL-12 and IL-23 subunits.

**Conclusion:**

The study concludes that poplar bud extract elicited antioxidant activity, antitumor properties on the breast cancer cell line, followed by an antiangiogenic effect and an immunomodulatory potential on human primary dendritic cells. The biological activity of *Populus nigra* L. buds extract may open new directions of research on the topic addressed.

## Background

*Populus nigra* L. (black poplar) belongs to the *Salicaceae* family, widespread especially in Europe and Asia. In Romania it grows through meadows and wetlands, but it can be also found in plains. Currently in therapy, the most used part of the tree are the foliar buds, due to the complex phytochemical composition responsible for a wide range of therapeutic activities. Poplar buds are described as conical, elongated, measuring 2 cm long and 5–8 mm thick, with a sharp and slightly curved tip. Poplar buds are covered with a sticky and shiny resin, having a weak balsamic and aromatic smell. On the central axis of the buds are 4–8 oval and sharp bracts, adherent due to the resins in the composition [1, 2].

Regarding the phytochemical composition of the vegetal product, flavonoid derivatives are currently described as one of the main phytochemicals. They are represented by flavones (chrysin, tectochrysin, apigenol), flavonols (galangin, kaempferol, quercetol), flavanones (pinocembrin, pinostrobin). Also, phenolic acids and their esters, such as caffeic acid, p-coumaric, isoferulic (over 17 different esters) are listed among the main phytochemicals detected in different poplar bud extracts. Beside the flavonoid derivatives and phenolic acids, extracts obtained from poplar buds represent a source of phenolic heterosides namely, populoside (the benzoic ester of salicoside) and salicoside [3, 4]. Different phytochemical studies have also reported the presence of other components in the composition of poplar bud extract. These phytochemicals include tannins, waxes, resins, glucose, fructose, oligosaccharides, triterpene derivatives (α and β amirenol) [5, 6]. Essential oils as well, mainly rich in cadinen, cineol, bisabolol, humulin, farnesol, bisabolene, have also been reported as components of black poplar bud extracts [7].

The vegetal product has been assigned with a wide range of properties both in folk and evidence-based medicine. Black poplar extracts have shown promising effects on patients with respiratory conditions, such as bronchitis, asthma, laryngitis, sore throat, and flu [8] due to expectorant, antimicrobial, anti-inflammatory, analgesic, anti-rheumatic, astringent, antioxidant and capillary-protective effects [9, 10]. In order to benefit from the maximum concentration of active principles, the harvest of the vegetal product is carried out in march-april, before the development of the leaves [11]. The resins from black poplar buds represents an important source of propolis [12]. An increased number of studies have proven that black poplar bud extracts represent a source of polyphenolic compounds responsible for the strong antioxidant properties. Moreover, literature includes studies which detail the antibacterial and antifungal activities of poplar bud extracts [13].

In addition, extracts obtained from this vegetal product present significant anti-inflammatory activity which is attributed to the high number of flavonoids (e.g. pinocembrin, quercetin). Using insulin-like growth factor 1 (HGF-1) cell lines Pobłocka-Olech et al. demonstrated that 20–40 μM of ethanolic poplar bud extract (Poland), significantly reduced the in vitro secretion of pro-inflammatory interleukins (IL-1β and IL-6) [14]. Wang et al., reported data about the anti inflammatory potential of *Populus x canadensis* buds (China), ethanolic extract [15]. Regarding the antitumor properties, literature is poor in studies that report this kind of activity. However, there are many phytochemicals which can be found in black poplar bud extracts which have been assigned with antitumoral effects (e.g. pinocembrin, chrysin, pinostrobin).

The aim of this study was to conduct a phytochemical characterization of black poplar bud extract obtained from the western areas of Romania, including the phenolic content, antioxidant screening along with a biological evaluation in terms of antimicrobial activity, in vitro anticancer effect (tested on MCF-7 breast cancer cell line), and immunomodulatory potential and an in vivo antiangiogenic effect.

## Materials and methods

### Plant materials and reagents

Poplar buds were collected from the west part of Romania (Timisoara) by a student of the Victor Babes University of Medicine and Pharmacy, identified in the department of Pharmacognosy, and assigned the voucher specimen code Pg 3/2019. The formal identification of the plant material was made by the coordinator of the discipline of Pharmacognosy, Prof. Dr. Habil. Pharm. Corina Danciu. The extraction procedure was as follows: 10 g of dried and grounded vegetable product was mixed with 100 mL of 70% ethanol and covered with parafilm. After 10 min at room temperature the extract was ultrasonicated for 30 min at the temperature of 50 °C and frequency of 40 KHz (FALC LBS 2 ultrasonic water bath). The extract was afterwards filtered through filter paper by the help of a vacuum pump (Vacuubrand). In order to eliminate the solvent, a rotary evaporator (HEIDOLPH Laborata 4000 efficient WB eco) was used at 50 °C, under reduced pressure. For better drying the extract was placed in an etuve (Genlab N40c) at 50 °C for 5–6 h. The extract (called on further in the paper- Pg) was subsequently stored at − 4 °C until use [10].

### Antioxidant activity (AOA) - radical scavenging activity

The AOA of each hydro-alcoholic Pg extracts was determined by DPPH (2,2-diphenyl-1-picrylhydrazyl, Sigma Aldrich, Germany) free radical scavenging assay, based on the method of Manzocco et al. [16] and modified by our research group [17]. To 0.5 mL of each hydro-alcoholic extract (50, 100, 250, 500 and 1000 μg/mL), 0.5 mL DPPH 1 mM ethanolic solution were added. The absorbance values were recorded spectrophotometrically, using a T70 UV/VIS Spectrophotometer (PG Instruments Ltd., Leicestershire, United Kingdom), in a continuous mode, for 20 min, against a blank at 517 nm. The oxido-reduction reaction between DPPH radical free and the antioxidants contained in the analyzed samples is considered to be finished after the change color occurs in the mixture (from intense purple to pale yellow). The results were expressed in comparison to an ethanol solution of ascorbic acid 400 μg/mL, used as standard reference. The inhibition percentage of free radical DPPH, was calculated using the following equation:

1 $$Inhibition\kern0.5em \left[\%\right]\kern0.5em =\kern0.5em \left[\left({A}_{DPPH}\kern0.5em -\kern0.5em {A}_{extract}\right)\kern0.5em +\kern0.5em {A}_{DPPH}\right]\kern0.5em \times \kern0.5em 100$$

where: A_DPPH_ – the absorbance of DPPH free radical, without extract, analyzed at 517 nm; A_extract_ - the absorbance of each extract with DPPH, analyzed at 517 nm.

The half maximum inhibitory concentration IC_50_ alongside with IC_10_, IC_20_, IC_80_ and IC_90_, were calculated using OriginPro 2020 software, by linear regression analysis curve plotting between inhibition percentage and concentration of the hydro-alcoholic extracts.

### Physicochemical screening

In order to characterize the compounds contained in Pg extract, Fourier-transform infrared spectroscopy and thermal analysis were employed.

#### Fourier-transform infrared spectroscopy - FT-IR

The FT-IR qualitative analysis [18], was employed to identify the functional groups of the main compounds, present in the dried Pg extract. The FT-IR spectra of each resultant compound were obtained using a Prestige-21spectrometer (Shimadzu, Duisburg, Germany), at room temperature conditions (22 °C ± 1 °C). The spectral region recorded the peaks in the range from 4000 to 400 cm − 1, with a peak resolution of 4 cm − 1, under reduced pressure, using KBr pellets.

#### Thermal analysis

Thermogravimetry-Differential scanning calorimetry (TG-DSC) is an analytical technique which was used to assess the stability and composition of organic compounds contained in dried extract based to Pg, thus predicting biological stability prior to in vitro and in vivo application [19]. TG-DSC analysis was performed using a STA 449C instrument (Netzsch, Selb, Germany) in air atmosphere at a flow rate of 20 mL/min. The curves were recorded in the range 25–1000 °C with a heating rate of 10 K/min, using alumina crucibles.

### In vitro antimicrobial effects

Another aim of this study was to screen this extract for its antimicrobial properties against eight microorganisms (Thermo Scientific, USA) (*Staphylococcus aureus*, ATCC code 25923; *Streptococcus mutans*, ATCC code 35668; *Streptococcus pyogenes*, ATCC code 19615; *Enterococcus faecalis*, ATCC code 51299; *Escherichia coli*, ATCC code 25922; *Pseudomonas aeruginosa*, ATCC code 27853; *Candida albicans*, ATCC code 10231; *Candida parapsilosis*, ATCC code 22019). Were selected these eight strains because they are the most common pathogens responsible for most of the healthcare problems. The susceptibility of microorganisms to the tested compounds was determined using disk diffusion method and dilution method, according to the European Committee on Antimicrobial Susceptibility Testing (EUCAST), Clinical Laboratory and Standard Institute (CLSI) and based on previous similar studies [20, 21].

#### Disk diffusion method

The antimicrobial activity of the extract was evaluated by the agar disk diffusion method, as previously described [22, 23]. The standardized suspension was prepared in normal saline to a concentration of 0.5 McFarland. The un-supplemented Mueller Hinton (MH) agar with 5% defibrinated horse blood and β-NAD (MH-F) was inoculated with 100 μL of this suspension. Were added 10 μL from each sample to a 6 mm diameter blank paper disk (BioMaxima, Lublin, Poland), placed on the surface of the MH or MH-F agar. After 24 h at 35–37 °C, we measure the inhibition areas in millimeters. For all microbial strains these assays were done in triplicate and the average values were registered. The antibacterial activity of this extract was established according to the standardized value of the positive control, for gentamycin a diameter of the inhibition area ≥ 15 mm was considered as susceptibility, while for fluconazole the diameter was ≥19 mm. For negative control, were used a disk impregnated only with dimethyl sulfoxide (DMSO).

#### Dilution method

Standardized suspension was adjusted, by dilution, obtaining a microbial suspension of approximately 10^5^ colony forming units (CFU)/mL. For each compound tested, serial dilutions were prepared in MH-F broth: 5, 2.5, 1.25, 0.625, 0.312, 0.156 mg/mL. One milliliter from each dilution of tested compound and 1 mL inoculum were added in six test tubes, to a final microbial suspension of 0.5 × 10^5^ CFU/mL and a final compound dilution between 2.5 and 0.078 mg/mL. After incubating at 37 °C for 24 h, the MIC was analyzed. To determine the MBC a volume of 1 μl from the test tubes with no visible growth was inoculated on Columbia agar supplemented with 5% blood. After incubation at 37 °C for 24 h, the lowest concentration that kills 99.9% of the microorganisms was established [21].

### Cell culture and cell preparation

The MCF-7 human breast adenocarcinoma (ATCC® HTB-22™) and MCF-10A human breast epithelial cells (ATCC® CRL-10317™) were purchased from the American Type Culture Collection (ATCC). MCF-7 cells were cultured in RPMI-1640 medium (ATCC® 30–2001™). The cell line was supplemented with 10% fetal bovine serum (FBS; Sigma-Aldrich, Germany) and 1% penicillin/streptomycin mixture (Pen/Strep, 10.000 IU/mL; Sigma-Aldrich, Germany). MCF-10A cells were cultured in Dulbecco's Modified Eagle Medium/Nutrient Mixture F-12 (DMEM:F12) supplemented with 20 ng/mL epidermal growth factor, 500 ng/mL hydrocortisone, 0.01 ng/mL insulin, 5% FBS and 1% Pen/Strep. The cells were maintained in standard conditions (humidified atmosphere with 5% CO_2_ and 37 °C). Human Dendritic cells were differentiated from isolated PBMCs from buffy coats according to Nair et al. [24]. In brief, density centrifugation was performed using Ficoll (GE Healthcare, Uppsala, Sweden). Isolated PBMCs have been plated in 6-well plates and supernatant has been discarded upon 2 h of plastic adherence. Subsequently, cells were differentiated in RPMI 1640 GlutaMax medium (Thermo Fisher Scientific, Massachusetts, USA) supplemented with 10% autologous serum, 100 IU/ml penicillin, 100 μg/ml streptomycin, 10 mM HEPES (Sigma–Aldrich, Steinheim, Germany), 1 mM sodium pyruvate and 50 μM 2-β-ME (Thermo Fisher Scientific, Massachusetts, USA) supplemented with 40 ng/ml recombinant human GM-CSF (Peprotech, NJ, USA) and human IL4 (Peprotech, NJ, USA) for 6 days. Differentiated cells were stimulated with the Pg extract in different concentrations (1–100 μg/ml) or 1% DMSO as a control. In parallel, 250 nM LPS stimulation was used to generate inflammatory dendritic cells. After stimulation, cells were harvested by cell scraping for further analysis and supernatant has been collected and snapfrozen for further analysis.

### Anticancer activity

#### Antiproliferative MTT assay

The Pg extract was evaluated for possible in vitro anticancer activity against the MCF-7 human breast cancer cell line. The effect of black poplar bud extracts on MCF-7 breast cancer cells viability was evaluated by means of MTT (3-(4,5-dimethylthiazol-2-yl)-2,5-diphenyltetrazolium bromide) assay. The method was conducted as previously described [25]. Briefly, 1 × 10^4^ cells/well were seeded in 96-well culture plates and allowed to adhere overnight. On the second day, the cells were stimulated with different concentrations of poplar bud extracts (10, 25, 50, 75, 100 and 150 μg/mL) and were incubated for 72 h. The Control group is represented by cells treated with the solvent DMSO (Sigma-Aldrich). After the 72 h incubation period, the cells were treated with 10 μL of 5 mg/mL MTT solution from the MTT kit (Sigma-Aldrich) and incubated for an additional 3 h. The obtained formazan crystals were dissolved in 100 μL of lysis solution provided in the MTT kit. The absorbance was determined at 570 nm with a microplate reader (BioRad, xMark Microplate Spectrophotometer).

#### Determination of the cytotoxic potential by lactate dehydrogenase (LDH) assay

The cytotoxic effect of Pg on MCF-7 tumor cells was determined by LDH assay (Cytotoxicity detection kit, 11644793001, Roche). Briefly, 5 × 10^3^ cells/well were seeded in 96-well culture plates and allowed to adhere overnight. The second day, the cells were stimulated with different concentrations of poplar bud extract (10, 25, 50, 75, 100 and 150 μg/mL) and incubated for 72 h. After the incubation period, 100 μL from each well was transferred into a 96-well culture plate and mixed with 100 μL of reaction mixture (prepared according to the manufacturer’s instructions) and incubated at room temperature for 30 min. The level of LDH release in the medium was measured at 490 nm and 680 nm using a microplate reader (BioRad, xMark Microplate Spectrophotometer). Untreated cells (low control) and cells treated with 1% (v/v) Triton- X100 (high control) were used to determine spontaneous and maximum release of LDH, respectively.

#### Scratch assay

The scratch assay was performed in order for the assessment of the regressive effect of Pg extracts on the invasion capacity of MCF-7 breast cancer cells. Several 2 × 10^5^ cells/well were seeded onto 12-well culture plates until 90% confluence was reached. After that, the attached cells were scratched following the diameter of the well using a sterile pipette tip. The detached cells and cellular debris were removed by gently washing the wells with Phosphate Buffer Saline (PBS). Furthermore, the cells were stimulated with different concentrations of poplar bud extracts (10, 25, 50, 75, 100 and 150 μg/mL). Wells were captured on images at 0 h and 24 h, in order to compare the cell growth of the stimulated and the control cells (no stimulation) in early stages and at consistent times. Images were taken with Olympus IX73 inverted microscope provided with DP74 camera (Olympus, Tokyo, Japan) and cellSense Dimension software was used for analyzing the cell growth. The scratch closure rate was calculated as previously described by Moacă et al. [26]:

2 $$\mathrm{Scratch}\ \mathrm{closure}=\left[\frac{A_{t0}-{A}_t}{A_{t0}}\right]\ast 100\;\mathrm{rate}$$

where: A_t0_ is the scratch area at time 0; A_t_ is the scratch area at 24 h.

#### DAPI (4′,6-diamidino-2- phenylindole) staining

In order to investigate the apoptotic potential of Pg at the selected concentration, MCF-7 cells were plated to an initial density of 5 × 10^5^ cells/well onto 6-well plates overnight. On the following day, the medium was removed, and the cells were stimulated with a fresh one containing the testing compound to a final concentration of 10, 25 50, 75, 100 and 150 μg/mL, respectively, for 72 h. At the end of the stimulation period, the medium was removed from the wells and the cells were washed with ice-cold PBS twice and were fixed with 4% paraformaldehyde in PBS, permeabilized with 2% Triton- X/PBS for 30 min, followed by a blocking step with 30% FCS/0.01% Triton-X. Finally, the cells were washed with PBS and stained with DAPI (300 nM) in a dark chamber for 15 min. Fluorescent images were taken at a magnification of 40X, with a fluorescence inverted microscope Olympus IX73, equipped with an integrated DP74 digital camera (Olympus, Tokyo, Japan).

#### Cell cycle analysis

To characterize cell cycle distribution, the deoxyribonucleic acid (DNA) content analysis of the cells was determined by FACSCalibur flow cytometer (Becton-Dickinson, Franklin Lakes, NJ, USA). The MCF-7 human breast cancer cells were seeded into 6 well plates and treated with poplar bud extracts. After 48 h the cells were collected and fixed with cold 70% ethanol (1000 μl) and stored for 30 min at 4 °C. After centrifugation (1500 RPM, 5 min), cold PBS was used to wash the cells. Afterwards, 50 μl of propidium iodide (BD Pharmingen, BD Biosciences) were added to the cells and then incubated for 10 min at 4 °C. The untreated cells and the cells treated with 0.15% DMSO were used as a control and solvent control respectively. As a final step the percentage of cells present in the different cell cycle (G0, G1, S, G2) phases was determined using Flowing software 2.5.1.

#### Annexin V-FITC apoptosis assay

The Pg extract was screened for the flow cytometric analysis with Annexin V-FITC apoptosis detection kit (Sigma-Aldrich) following the manufacturer’s protocol. A number of 10^4^ cells/well were seeded into a 6-well plate and left overnight. After 24 h, the media was removed and were added fresh medium treated with Pg extracts (10, 25, 50, 75, 100, 150 μg/mL). After 72 h the cells were trypsinized and were washed two times with Binding Buffer, centrifuged at 1500 RPM for 7 min, resuspended in Binding Buffer and incubated with 5 μL of Annexin V-FITC for 15 min at room temperature. After cell washing, the cell pellet was resuspended in a 190 μL Binding Buffer with 10 μL PI solution (propidium iodide). As control was used the untreated cells, and as a solvent control was used cells treated with 0,15% DMSO. Cells were analyzed by flow cytometry (FACSCalibur, Becton Dickinson) using the fluorescence channel FL1 for annexin and FL2 for PI. The results were analyzed using Flowing Software 2.5.1.

### Angiogenesis evaluation using the chick chorioallantoic membrane (CAM) assay

In order to investigate the effects of the Pg on the process of angiogenesis we conducted an in vivo experiment using as biological material the chorioallantoic membrane of fertilized chicken (*Gallus gallus domesticus*) eggs under development. The eggs were incubated at 37 °C and 50% humidity and were prepared according to the basic protocol with some modifications [25, 27]. On the third day of incubation 5–6 ml of albumen were removed, and subsequently an opening was cut on the upper side of the eggs. The eggs were further incubated until day 7 of incubation, when 10 μl of test (Pg 150 μg/mL) and control (0.1% DMSO v/v in double distilled water) samples were inoculated inside plastic rings on the developing vascular plexus of the CAM. Application of the samples was repeated daily (24 h, 48 h) and was followed by stereomicroscopic monitoring, capturing relevant *in ovo* images (Discovery 8 Stereomicroscope, Zeiss, coupled to Axio CAM 105 color, Zeiss digital camera). The photographs were further processed by Zeiss ZEN software, ImageJ and GIMP.

### Immunomodulatory activity

#### Cell viability and apoptosis assay

Cytotoxic effects of the Pg extract on dendritic cells were analyzed by FACS staining with DAPI for cell viability and Annexin V/7AAD for apoptosis.

Additionally, to the stimulation of the differentiated inflammatory dendritic cells with the Pg extract in different concentrations, 1 μM Staurosporin (LC Laboratories, MA, USA) was used as a positive control for apoptosis. After 24 h cells were harvested and washed twice with Annexin Binding Buffer (0.1 M NaCl, 25 mM CaCl2, 0.1 M Hepes), resuspended in 500 μl Annexin Binding Buffer and stained with 5 μl Annexin V FITC (Immunotools, Friesoythe; Germany) per tube for 15 min in the dark. After this incubation 5 μl of 7AAD (Invitrogen, MA, USA) was added and stained for further 5 min in the dark. The cells were pelleted and resuspended in 400 μl Annexin Binding Puffer containing 100 ng/ml DAPI (Sigma, Merck, Darmstadt) for cell viability staining. During the next hour the samples were measured using FACS Canto II, the data were analyzed with FlowJo software 7.6.5.

#### Cytokine analysis

The supernatants were analyzed by ELISAs for IL-10 and IL-23 (R&D Systems, Wiesbaden, Germany) according to the manufacturer’s manual. The cell pellet was harvested, and total RNA was extracted using the peqGOLD Total RNA Kit (peqlab, Erlangen, Germany) as recommended by the manufacturer. RNA concentration was measured using the Nano-Drop 1000 analyzer (Thermo Scientific) and adjusted to 1 μg/μL for cDNA synthesis using the high-capacity cDNA reverse transcription kit (Life Technologies, Carlsbad, CA). TaqMan® gene expression assays (Thermo Fisher, Dreieich, Germany) were applied for all genes of interest and for the housekeeping genes GAPDH (Glyceraldehyde 3-phosphate dehydrogenase) andFBXO38, (Primer Design, Southampton, UK). The Precision FAST Mastermix (Primer Design) was used, and quantitative real-time PCR was run according to manufactures recommendations (7500 Fast Real-Time PCR System, Applied Biosystems). Data were evaluated using the mean of the two housekeeping genes as a reference.

### Statistical analysis

The results obtained in the in vitro assay are expressed as mean ± standard deviation. Comparison among groups was made using One-way ANOVA test and Dunnett’s multiple comparison post-test. For the statistical analysis GraphPad Prism 5 (GraphPad Software, San Diego, CA, USA) was used.

## Results

### Antioxidant activity

**Fig. 1:**
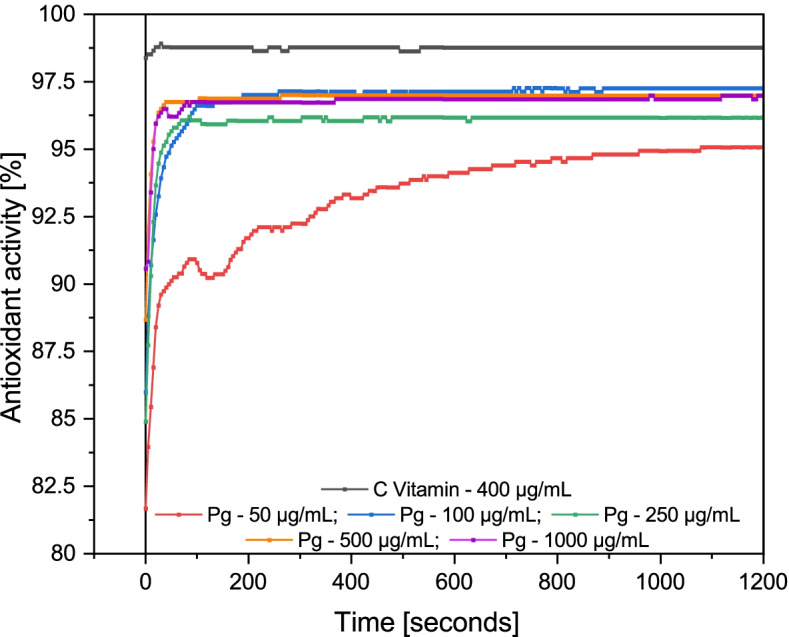
AOA of Pg extracts (50, 100, 250, 500, 1000 μg/mL) compared to ascorbic acid (400 μg/mL)

The antioxidant activity of the hydroalcoholic extract obtained from Pg was determined spectrophotometrically, by radical scavenging 2,2-diphenyl-1-picrylhydrazyl (DPPH) assay using Ascorbic acid as a standard reference. Table 1 shows the percentage of inhibition obtained after evaluating the Pg hydroalcoholic extracts as a function of their concentration. It can be seen that the five concentrations of tested Pg extracts have very high antioxidant activity, almost comparable to the AOA of the standard reference (vitamin C ethanolic solution). Both the antioxidant activity of Pg extracts and the AOA of different concentrations of vitamin C, expressed as a percentage, were calculated with eq. (1), presented above. The analyzes were performed three times, for each hydroalcoholic extract, respectively each concentration of vitamin C separately, thus calculating the standard deviation. The mean of the AOA obtained for each analyzed sample was used to determine the IC_50_ values, IC_10_, IC_20_, IC_80_ and IC_90_, respectively, presented in Table 2**.** The percentage of inhibition in the case of the analyzed ethanolic Pg extract varies from 95 to 97.3%, while in the case of vitamin C, between 97.5–98.9%. As already well known IC_50_ is the average inhibitory concentration (represents the maximum concentration of the hydroalcoholic extract of Pg, necessary to inhibit 50% of the standard oxidant used DPPH), an operational value, depending on the test conditions. All determined inhibitory concentrations, expressed in Table 2, IC_50_ as well as IC_90_ - the inhibitory concentration required to inhibit 90% of the standard oxidant, were determined to establish complete inhibition. Assuming the binding of DPPH to the antioxidants contained in the Pg extract, in a single place at equilibrium, with a Hill coefficient equal to 1, IC_90_ should have been 10 times higher than IC_50_ [28]. But for a competitive inhibitor (such as Pg ethanolic extract), IC_50_ increases with increasing concentration. Figure 1 shows the reaction kinetics, analyzed for 1200 s for each concentration of Pg ethanolic extract mixed with DPPH, compared to the reaction kinetics between vitamin C and DPPH. From the graph it can be seen that Pg extract analyzed at the lowest concentration (Pg 50 μg/mL) reacts slowly with the DPPH radical, the reaction reaching equilibrium only after 1100 s. As the lowest concentration tested over time reacts with the standard oxidant, it confirms that the Pg ethanolic extract is a competitive inhibitor of vitamin C, which is a non-competitive inhibitor, consuming the entire amount of DPPH in the first 20 s from the start of the reaction. The fact that vitamin C is a non-competitive inhibitor also results from the fact that the same value of the inhibition percentage was obtained both at the concentration of 25 μg/mL as well as at the concentration of 200 μg/mL concentration. The rest of the screened Pg concentrations reached faster the equilibrium (interval between 100 and 400 s, depending on the concentration), consuming the entire amount of DPPH after this interval. The fact that Pg extract is a competitive inhibitor also results from the small fluctuations observed in Fig. 1, recorded until the redox reaction reached equilibrium.

**Table 1:** The percent of AOA induced by vitamin C as compared to Pg extracts

Vitamin C		***Pg*** extracts	
Conc. [μg/mL]	% inhibition	Conc. [μg/mL]	% inhibition
**25**	**98.90561 ± 0.0009**	**50**	**95.07524 ± 0.0039**
**50**	**97.53762 ± 0.0019**	**100**	**97.26402 ± 0.0022**
**100**	**98.49521 ± 0.0012**	**250**	**96.16963 ± 0.0030**
**200**	**98.90561 ± 0.0009**	**500**	**96.99042 ± 0.0024**
**300**	**97.81112 ± 0.0017**	**1000**	**96.99042 ± 0.0024**
**400**	**98.76881 ± 0.0010**

**Table 2:** The concentrations of the inhibitors (vitamin C and Pg extracts) where the response is reduced by half (IC_50_), at 10% response (IC_10_), at 20% response (IC_20_), at 80% response (IC_80_) and at 90% response (IC_90_)

	IC_**50**_ [μg/mL]	IC_**10**_ [μg/mL]	IC_**20**_ [μg/mL]	IC_**80**_ [μg/mL]	IC_**90**_ [μg/mL]
**Vitamin C**	**2.26599 ± 3.16**	**2.39958 ± 5.10**	**2.34938 ± 4.36**	**2.18555 ± 2.05**	**2.13938 ± 1.43**
***Pg*** **extract**	**0.36431 ± 0.52**	**0.10804 ± 0.26**	**0.16921 ± 0.34**	**0.78436 ± 0.66**	**1.2284±** **0.61**

### Physicochemical screening

#### Fourier-transform infrared spectroscopy (FT-IR) spectroscopy

**Fig. 2:**
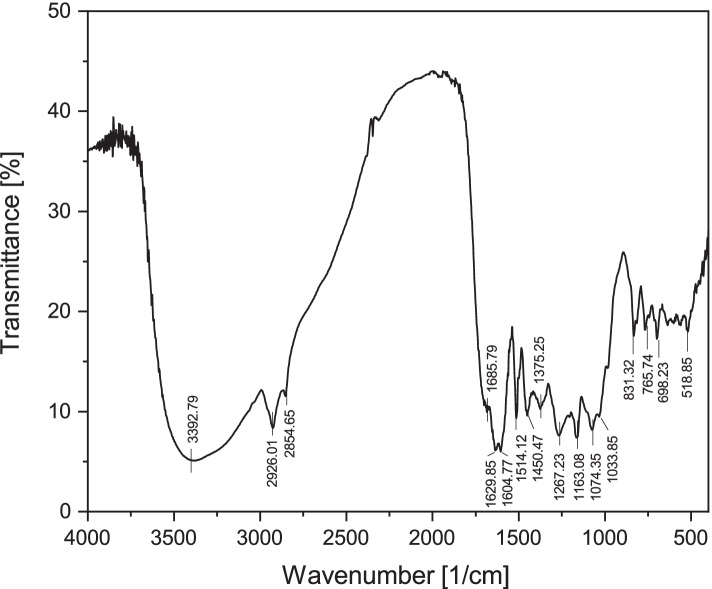
FT-IR spectra of Pg extract

In Fig. 2 and Table 3 are given the results of FT-IR analysis of Pg dried extract. The interpretation of the FT-IR spectra was performed in accordance with the Characteristic IR Absorption Frequencies of Organic Functional Groups [29].

**Table 3:** Peak values and functional groups of Pg dried extract in the FT-IR spectra

Characteristic absorptions [cm^**− 1**^] leaves extract / stems extract	Functional group	Bond	Intensity
3392.79	Alcohol	O-H stretch (H-bonded)	Strong, broad
2926.01	Alkanes	C-H stretch	Strong
2854.65	Aldehyde	=C-H stretch	Medium, two peaks
1685.79 / 1629.85	Alkene	C=C stretch	Variable
1604.77	Aromatics	C=C stretch	Medium-weak, multiple bands
1514.12 / 1375.25	Nitro	N-O stretch	Strong, two bands
1450.47	Alkane	C-H bending	Variable
1267.23	Carbonyl acids	C-O stretch	Strong
	Amine	C-N stretch	Medium - weak
1163.08	Amine	C-N stretch	Medium - weak
1074.35 / 1033.85	Alcohol	C-O strecth	Strong
	Ester	C-O stretch	Two bands or more
831.32 / 765.74 / 698.23	Alkene	=C-H bending	Strong
	Alkyl Halide	C-Cl stretch	Strong
518.85	Alkyl Halide	C-Br stretch	Strong

As can be seen, FT-IR spectra had some relevant absorption peak at, 2926.01 cm^− 1^, 1629.85 and 1604.77 cm^− 1^, 1267.23 cm^− 1^ and 1074.35 cm^− 1^. The peak identified at 3392.79 cm-1, points out a broad band usually assigned to the stretching vibration of O–H from hydroxyl groups (H - bonded) and water. Hydroxyl groups may belong to the phenolic acid, phenolic glycosides or tannins contained in the Pg dried extract. The peak situated at 2926.01 cm^− 1^ corresponds to the saturated aliphatic C-H bands. Saturated aliphatic C-H bands are due to the presence of tannins in the analyzed extract [30]. The peaks at 1629.85 and 1604.77 cm^− 1^ may be assigned to stretching vibration of C=C bands. This C=C band confirms either the presence of alkene functional group, or the presence of aromatic functional group. Most probable, this band is assigned to the aromatic functional group, due to the medium-weak intensity of the absorption peak and to the multiple bands recorded.

The band located at 1267.23 cm^− 1^ is assigned to stretching the vibration of C-O bands. This C-O band confirms the presence of a carbonyl acid as well as the presence of ethers in the analyzed extract. Could also be the presence of an ester, but we rule out the possibility due to the fact that regarding the intensity, esters should present two bands or more recorded on the spectra. Whereas the band located at 1267.23 cm^− 1^ is not so broad, rather is medium-weak intensity with a stretch vibration type. We believe that in the analyzed extract, amine groups are also present like C-N bands (phenolic acids from Pg dried extract), also confirmed by the presence of the band from 1163.08 cm^− 1^ with a medium intensity. The band located at 1074.35 cm^− 1^ is assigned to a stretching vibration of C-O bands, present in alcohol functional groups (monoterpenes and non-terpenes contained in the extract) and/or to a stretching vibration of C-O bands, present in ester functional group, due to the appearance of the second bands on FT-IR spectra (1033.85 cm^− 1^). The flavonoids/flavonols present in the Pg dried extract contain ester-like functional groups in their structure. The last three bands highlighted on the FT-IR spectra (765.74; 698.23 and 518.85 cm^− 1^) may be due to both the present of alkene functional groups (765.74 and 698.23 cm^− 1^– assigned to a bending vibration of = C-H bands, belonging to the aldehides from Pg dried extract) as well as alkyl halide functional groups, assigned to a stretching vibration of C-Cl bands. As in the case of the two bands described above, the band located at 518.85 cm^− 1^ (assigned to C-Br bands from the alkyl halide functional groups) the presence of these compounds may be due either the presence of some minerals that have been absorbed by buds from the soil through the poplar (concentrating over time), or due to the impurity of the extract at preparation time or even at characterization time. All the absorption peaks recorded following the FT-IR analysis as well as functional groups together with characteristic absorption band and intensity, are given in Table 3.

#### Thermal analysis

**Fig. 3:**
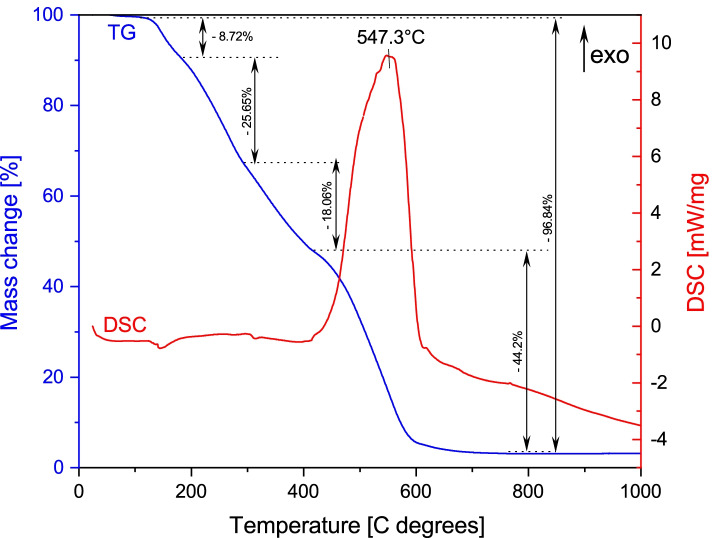
TG-DSC curves of Pg (aqueous, alcoholic, hydro-alcoholic) extract

Thermogravimetry- Differential Scanning Calorimetry (TG-DSC) graphic of dried Pg extract is presented in Fig. 3. As can be seen the thermal analysis conducted to the fact that the total mass loss of 96.84% in four stages. In the first stage, the lowest mass loss occurs (8.72%) of the total mass without energy changes, noticeable on the DSC curve. The same thing happens both in the second stage, located between 150 and 300 °C, as well as in the third stage (300–400 °C); the only recorded effects on the TG curve are weight changes (25.65% - on the second stage and 18.06 on the third stage). In the fourth stage of Pg extract degradation, located between 400 and 800 °C it takes place the largest mass loss (44.2%), accompanied by an exothermic process with a maximum at 547.3 °C. After that, the complete degradation of the extract occurs, because no mass changes on TG curve wererecorded. The exothermic effect noticed on the DSC curve, could be related with the degradation of aromatic compounds, carbohydrates and aromatic amino acids present in the dried extract of Pg.

### In vitro antimicrobial effects

The Gram-negative bacilli strains, especially *Escherichia. coli* and *Pseudomonas aeruginosa* presented significant inhibitory activity at the tested concentrations. The inhibition diameters are presented in Table 4. Table 5 presents the results on the minimum inhibitory concentration (MIC) and Minimum bactericidal concentration (MBC) values. The MIC values for *Streptococcus. pyogenes* and *Streptococcus. mutans***.** (0.312 mg/mL) were lower than those for *Staphylococcus aureus* (0.625 mg/mL) and *E. faecalis* (2.5 mg/mL). The MBC values were very appropriate to the MIC values. Based on these results, we can affirm that screened Pg extracts present bactericidal effects only against the Gram-positive cocci and Candida spp. The extract does not have any activity on *Pseudomonas aeruginosa* and *Escherichia coli* strains.

**Table 4:** The inhibition diameters for selected strains after incubation with Pg

*Compound*	*Streptococcus pyogenes*	*Streptococcus mutans*	*Staphylococcus aureus*	*Enterococcus faecalis*	*Escherichia coli*	*Pseudomonas aeruginosa*	*Candida albicans*	*Candida parapsilosis*
PG	23 mm	26 mm	15 mm	13 mm	7 mm	8 mm	17 mm	17 mm

**Table 5:** The minimum inhibitory concentration (MIC) and the minimum bactericidal concentration (MBC)

Species	MIC (mg/mL)	MBC (mg/mL)
*S. pyogenes*	0.312	0.312
*S. mutans*	0.312	0.312
*S. aureus*	0.625	2.5
*E. faecalis*	2.5	–
*E. coli*	–	–
*P. aeruginosa*	–	–
*C. albicans*	1.25	2.5
*C. parapsilosis*	1.25	2.5

### Anticancer activity

#### MTT(3-(4,5-dimethylthiazol-2-yl)-2,5 diphenyl tetrazolium bromide) assay

**Fig. 4:**
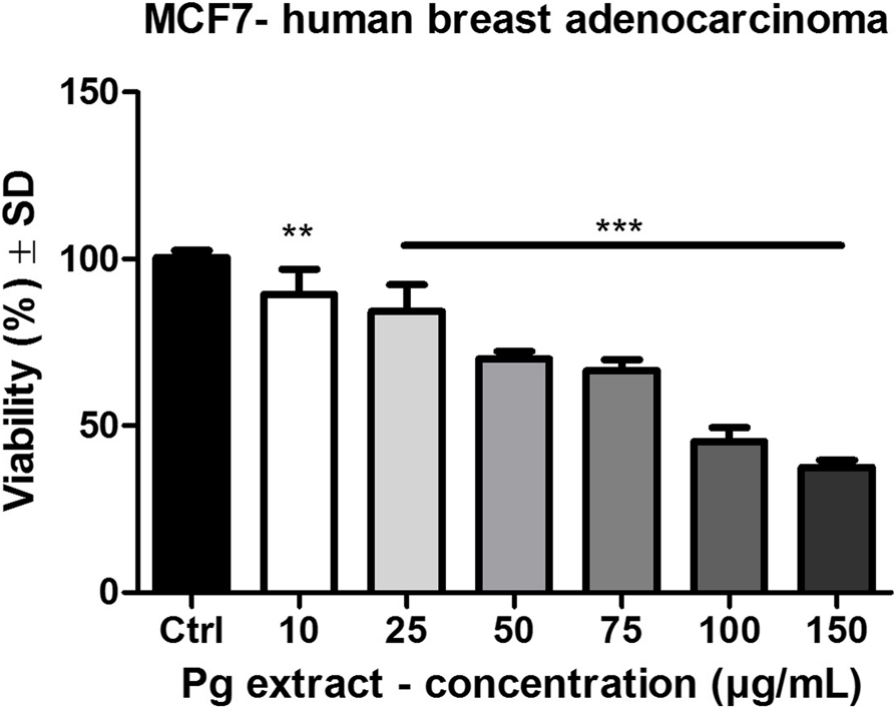
MCF-7 human breast adenocarcinoma cells viability after 72 h stimulation with Pg (10, 25, 50, 75, 100 and 150 μg/mL). The results are expressed as cell viability percentage (%) related to the Control cells. Comparison among groups was made using One-way ANOVA test and Dunnett’s multiple comparison post-test. (** *p* < 0.01; *** *p* < 0.001 vs. Control)

The effect of Pg extract was assessed on MCF-7 human breast adenocarcinoma cell lines and compared to the Control group, which are cells that were stimulated with the solvent DMSO. Also, a non-tumorigenic cell line was used (MCF-10A breast epithelial cells) in order to demonstrate the selectivity of the screened samples on cancer cells. In Fig. 4 is represented the effect of Pg extract on MCF-7 human breast adenocarcinoma cells after a stimulation period of 72 h. The IC_50_ value of Pg extracts on MCF-7 human breast adenocarcinoma cells after 72 h post-stimulation was 66.26 μg/mL. Treatment with Pg extract elicited a dose-dependent decrease of tumor cell viability. At the highest tested dose, tumor cells viability was decreased to 37.5 ± 2% vs. Control.

**Fig. 5:**
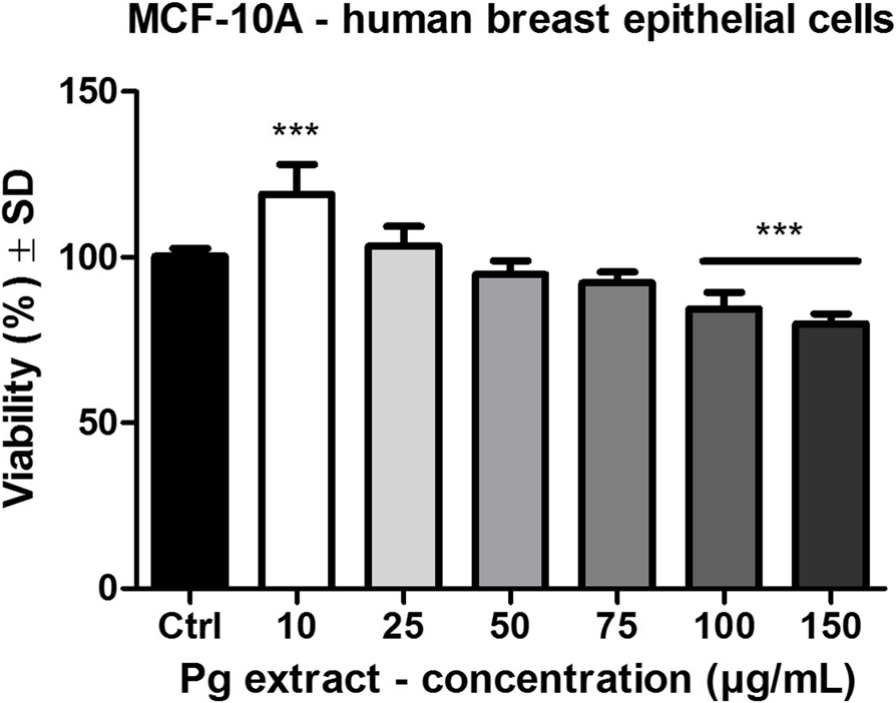
MCF-10A human breast epithelial cells viability after 72 h stimulation with Pg (10, 25, 50, 75, 100 and 150 μg/mL). The results are expressed as cell viability percentage (%) related to the Control cells. Comparison among groups was made using One-way ANOVA test and Dunnett’s multiple comparison post-test. (*** *p* < 0.001 vs. Control)

Figure 5 depicts the effect of Pg extract on MCF-10A breast epithelial cells after 72 h stimulation. It can be observed that at the lowest dose tested (10 μg /mL), the extract produced a significant increase in cells viability (119.03 ± 9% vs. Control). Only at the highest doses tested a decrease in cells viability was noticed (for 100 μg /mL cells viability was 84.4 ± 4.9% vs. Control and for 150 μg/mL cells viability was 79.8 ± 3% vs. Control). The results obtained indicate that Pg extract is selective on the screened cancer cell line, affecting more the breast tumor cells than the healthy cell line.

#### Determination of the cytotoxic potential by lactate dehydrogenase (LDH) release

**Fig. 6:**
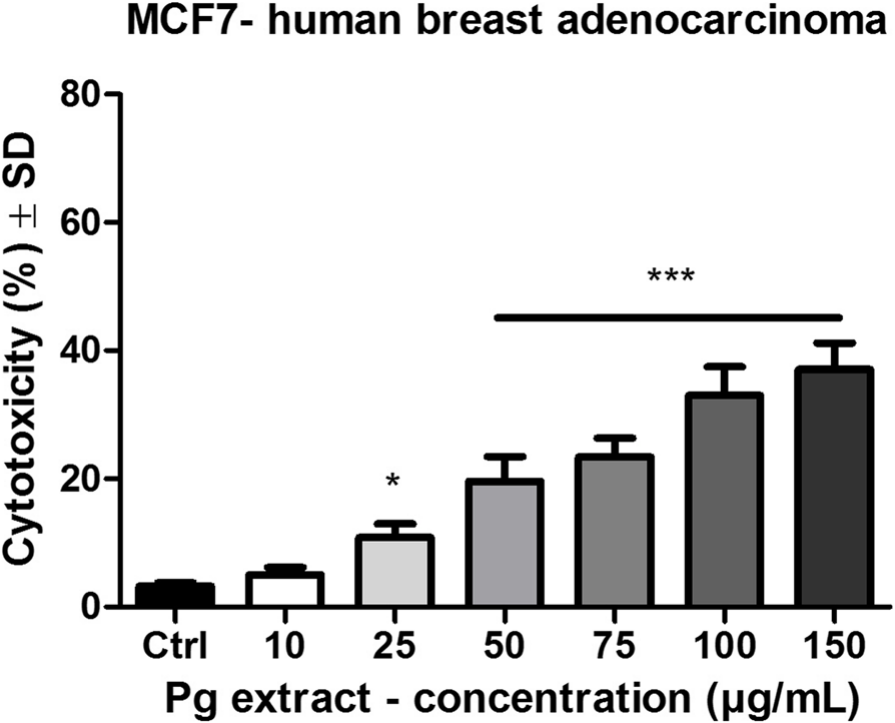
The cytotoxic effect of Pg (10, 25, 50, 75, 100 and 150 μg/mL) on MCF-7 human breast adenocarcinoma cells after 72 h stimulation. The results are expressed as cytotoxicity percentage (%) related to the Control cells. Comparison among groups was made using One-way ANOVA test and Dunnett’s multiple comparison post-test. (* *p* < 0.05; *** *p* < 0.001 vs. Control)

In order to evaluate the cytotoxic potential of Pg extract at the selected concentrations, lactate dehydrogenase assay was performed. After the 72 h stimulation period of MCF-7 cells a dose dependent increase in the release of lactate dehydrogenase was observed (Fig. 6), indicating that the extract produced a cytotoxic effect on the cancer cell line. At the highest dose tested (150 μg/mL) the cytotoxic rate was 37 ± 4.1% vs. Control (5 ± 1.1%).

#### Scratch assay

**Fig. 7:**
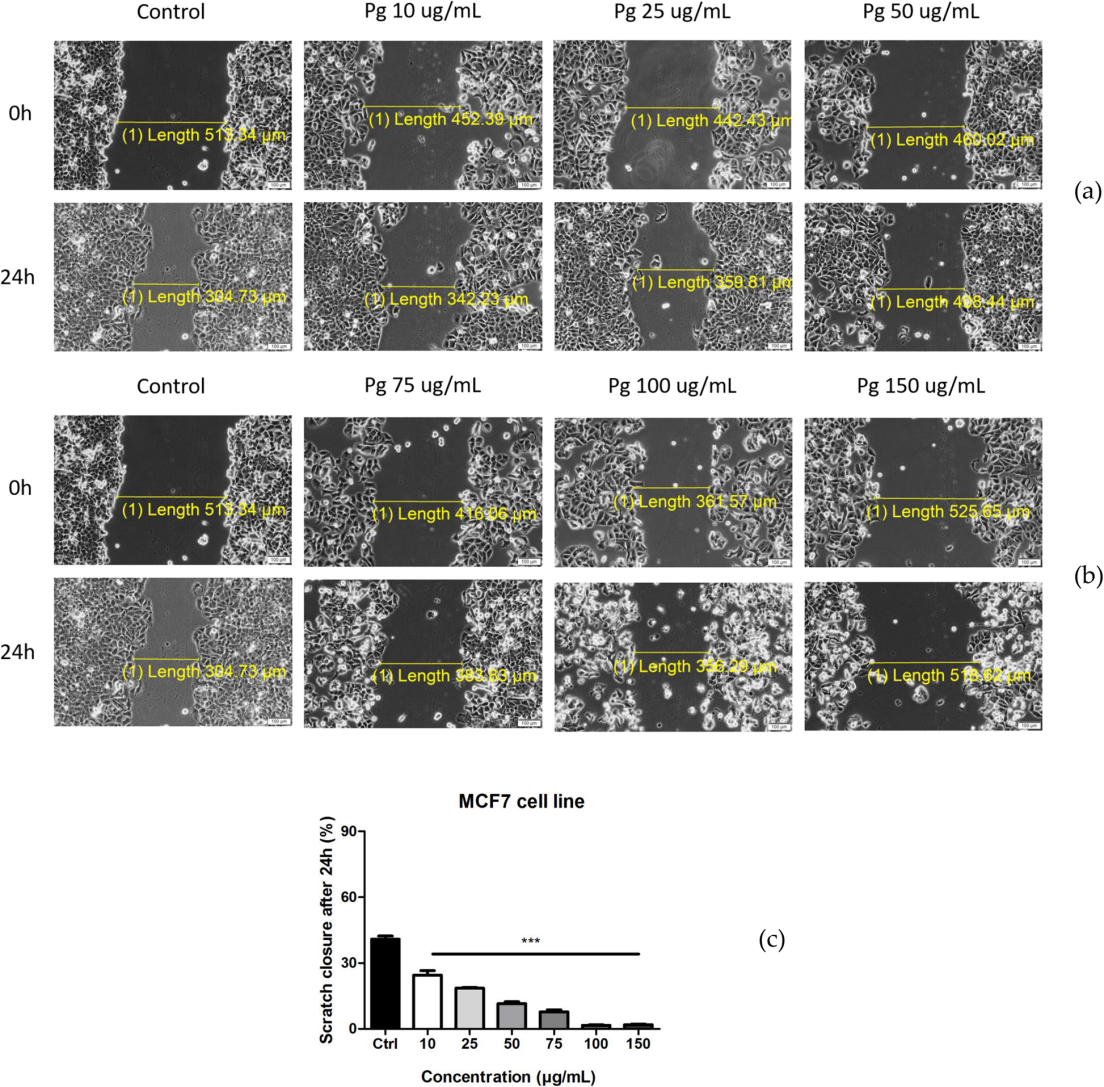
**a** Pg extract (10, 25, and 50 μg/mL) activity on MCF-7 human breast adenocarcinoma cells migration and proliferation potential. Progression of cell migration was monitored by imaging the scratch line initially and at 24 h post-stimulation. Images were taken by light microscopy at 10× magnification; **b** Pg extract (75, 100, and 150 μg/mL) activity on MCF-7 human breast adenocarcinoma cells migration and proliferation potential. **c** The anti-migratory potential of Pg extract (10, 25, 50, 75, 100 and 150 μg/mL) on MCF-7 breast adenocarcinoma cells. The bar graphs are expressed as percentage of scratch closure after 24 h compared to the initial surface. Comparison among groups was made using One-way ANOVA test and Dunnett’s multiple comparison post-test. (*** *p* < 0.001 vs. Control)

On MCF-7 human breast adenocarcinoma cells the Pg extract inhibited cell migration and proliferation in a dose dependent manner (Fig. 7a, b). Furthermore, at the highest tested doses (100 and 150 μg/mL) Pg extract also generates changes in tumor cell morphology and some of the cells start to be detached.

In Fig. 7c) is depicted the Scratch closure rate for all the concentrations. It can be observed that Pg extract produced a dose-dependent decrease in the closure rate showing that the sample elicited an anti-migratory effect on MCF-7 breast adenocarcinoma cells. At the highest doses tested doses the scratch closure rate was 1.4% for 100 μg/mL and 1.3% for 150 μg/mL.

#### DAPI (4′,6-diamidino-2- phenylindole) staining

**Fig. 8:**
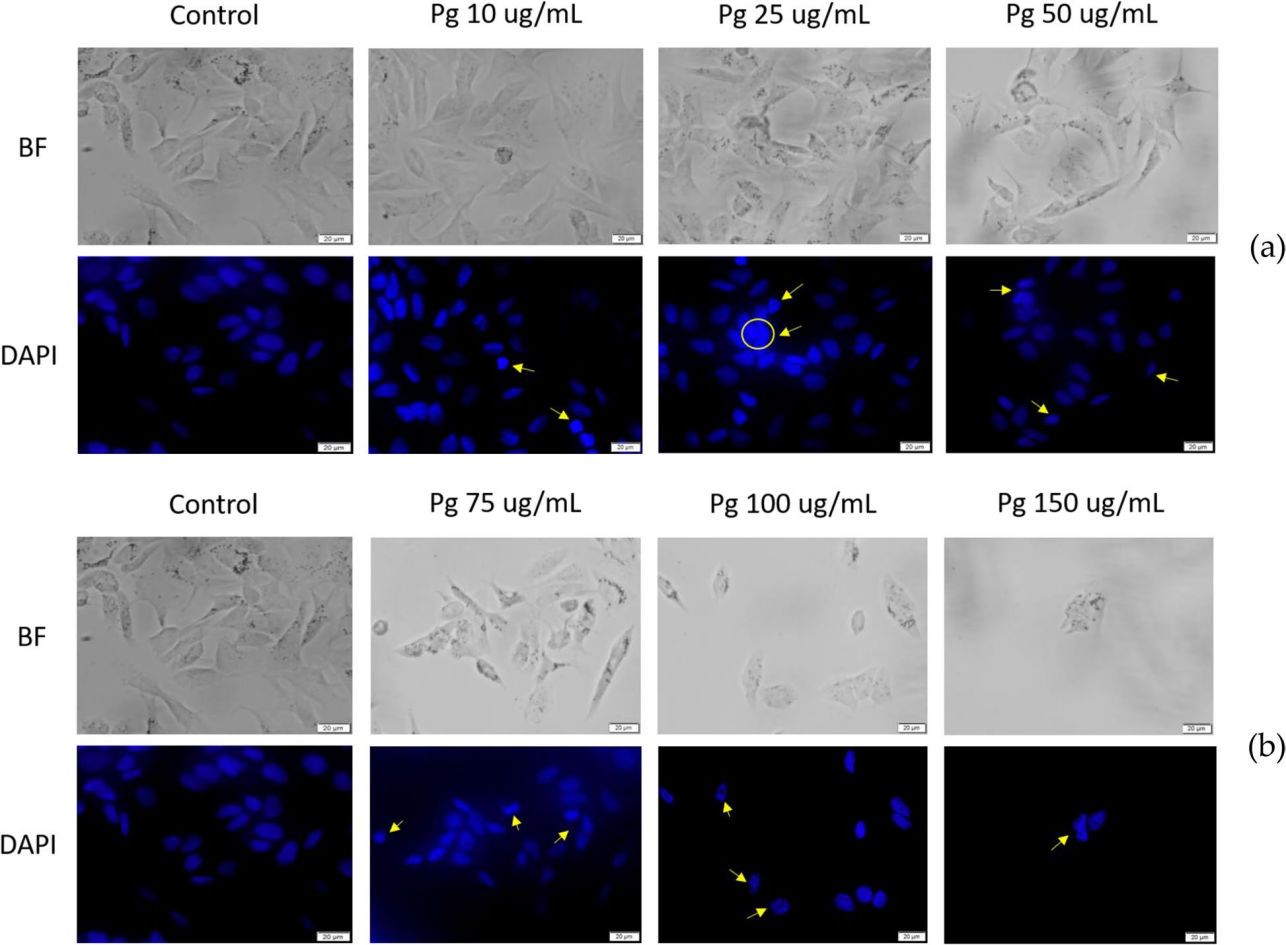
MCF-7 human breast adenocarcinoma cells treated with Pg extract at different concentrations (**a**) (10, 25, and 50 μg/mL) and (**b**) (75, 100, and 150 μg/mL) - for 72 h - BF = bright field microscopy; DAPI staining was performed for apoptotic morphological characteristics

Figure 8 depicts the DAPI staining of MCF-7 breast adenocarcinoma cells: the images show that chromatin condensation is increasing dose-dependent and that there are signs of nuclear membrane blebbing. These results indicate that there are signs of apoptosis in MCF-7 cells following treatment with Pg extract. DNA fragmentation was not observed under the current experimental conditions. In the Control cells the chromatin density was equally dispersed.

#### Cell cycle arrest

**Fig. 9:**
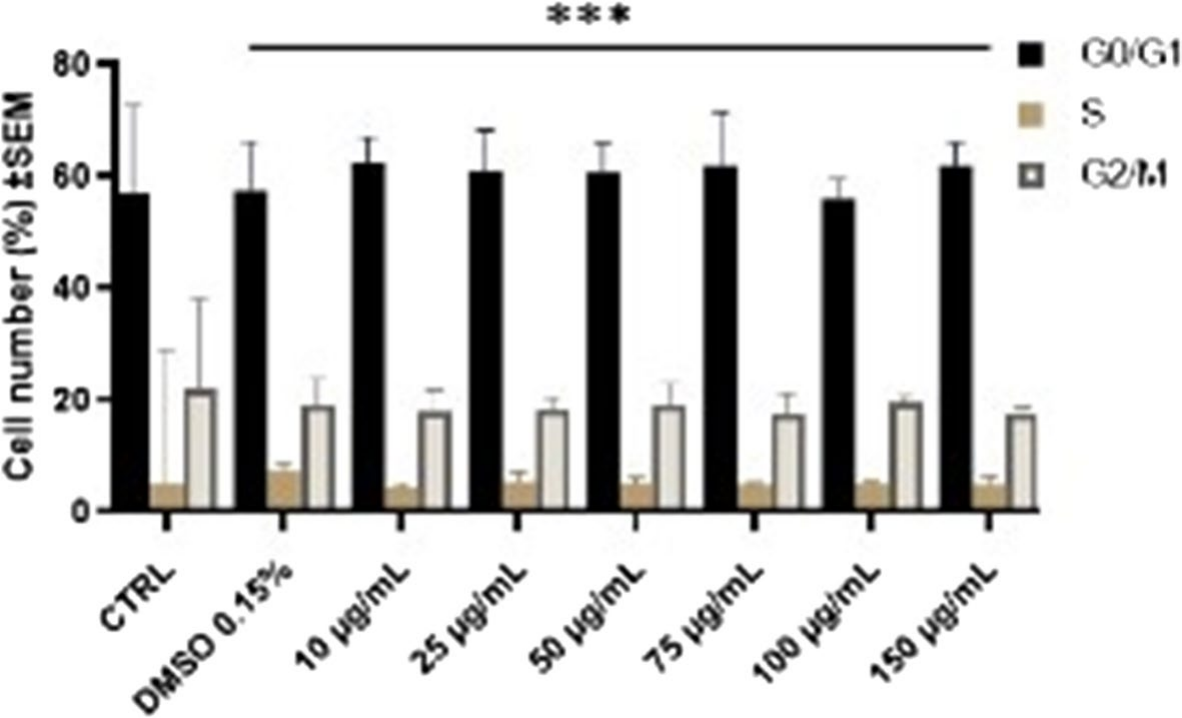
MCF-7 human breast adenocarcinoma cell cycle arrest after stimulation with Pg (10, 25, 50, 75, 100 and 150 μg/mL) on G0/G1; S and G2/M phases. The results are expressed as cell viability percentage (%) related to the Control cells. Comparison among groups was made using One-way ANOVA test and Dunnett’s multiple comparison post-test. (*** *p* < 0.0001 vs. Control)

To better understand the mechanism of inhibition of cell proliferation, the distribution of cells in the different phases of the cell cycle was analyzed following treatment with 10, 25, 50, 75, 100, 150 μg/mL Pg for 72 h. A slight increase of the cells in G0/G1 phase with a concomitant decrease in cells in the G2/M phase was observed, but the G0/G1 arrest was not in a dose-dependent manner, the percentage of cells in G0/G1 phase increasing from 56.97 ± 8.93% (control) up to 62.46 ± 11.93 (10 μg/mL) or 61.96 ± 4.05% (150 μg/mL). Figure 9 presents the results of all cell cycle experiments.

#### Annexin V-Propidium iodide (PI)

**Fig. 10:**
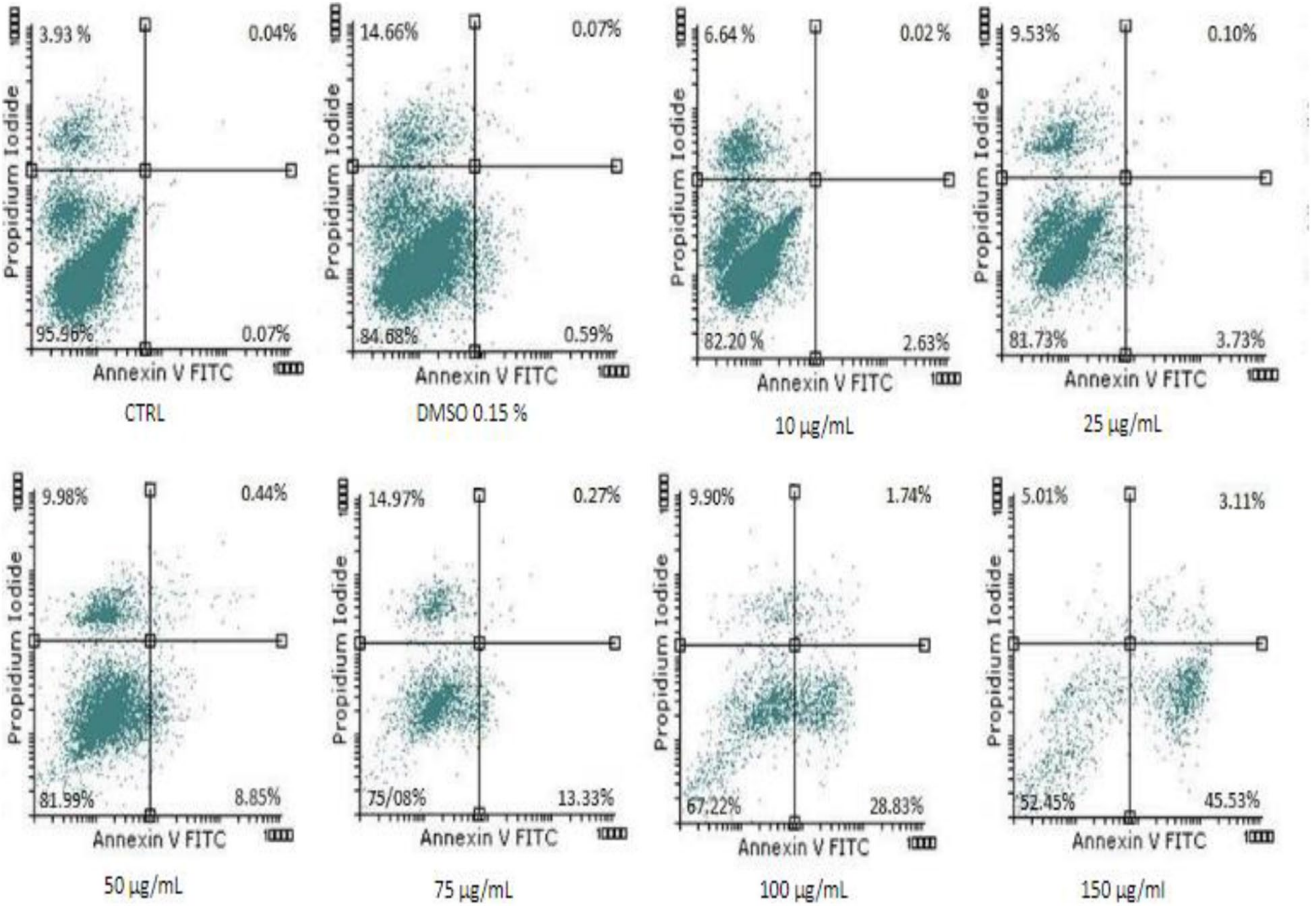
Representative histograms. Viability of MCF-7 human breast cancer cell line using Annexin V/PI analysis

Annexin-PI double staining assay is a commonly used approach for achieving information regarding phenomena of early apoptosis, late apoptosis and necrosis. The representative flow-cytometry dot-plots for the cells treated with different concentrations of Pg (10, 25, 50, 75, 100, 150 μg/mL), control and DMSO are presented in Fig. 10.

Lower right quadrant represents early apoptotic cells while upper right quadrant and upper left quadrant represent late apoptotic cells and necrotic cells respectively. One can notice that Pg at the maximum concentration tested (150 μg/mL) caused early apoptotic events in a dose-dependent manner when compared to control (0.07% ± 0.05% vs. 45.53% ± 6.5%). One can notice that Pg had an apoptotic effect on MCF-7 cells in a dose-dependent manner, inducing early apoptosis and late apoptosis. The percentage in early apoptotic cells after 72 h of treatment with Pg extract, increased dramatically from 0.07% ± 0.05 in case of untreated cells, up to 45.53% ± 6.50 in case of 150 μg/mL.

**Fig. 11:**
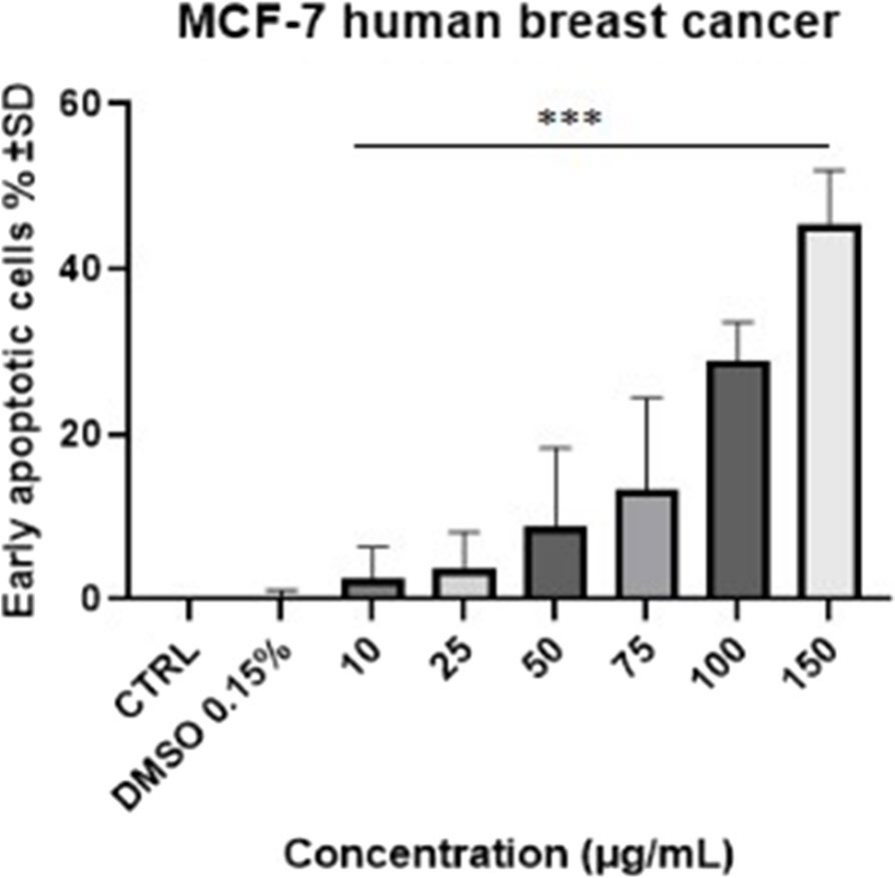
The pro-apoptotic potential of Pg extract (10, 25, 50, 75, 100 and 150 μg/mL) on MCF-7 breast adenocarcinoma cells. The bar graphs are expressed as the percentage (%) of early apoptotic cells related to control cells. Comparison among groups was made using One-way ANOVA test. (*** *p* < 0.001 vs. Control)

As shown in Figs. 10 and 11 lower concentrations had a proapoptotic effect, with the percentage of early apoptotic cell as follow: the lowest concentration (10 μg/mL) lead to an increase of 2.63% ± 3.72, while 25, 50, 75 and 100 μg/mL lead to an increase of 3.73% ± 4.49, 8.85% ± 9.55, 13.33% ± 11.12, 28.83% ± 4.68, respectively (*p* < 0.001). Regarding the late apoptosis, a slight increase in late apoptotic cells was observed starting with the concentration of 50 μg/mL (0.44% ± 0.32 compared with 0.04% ± 0.03 for control) increasing progressively up to 1.74% ± 0.25 and 3.11% ± 3.08 for 100 and 150 μg/mL, respectively (*p* < 0.01).

### Chorioallantoic membrane (CAM) assay

**Fig. 12:**
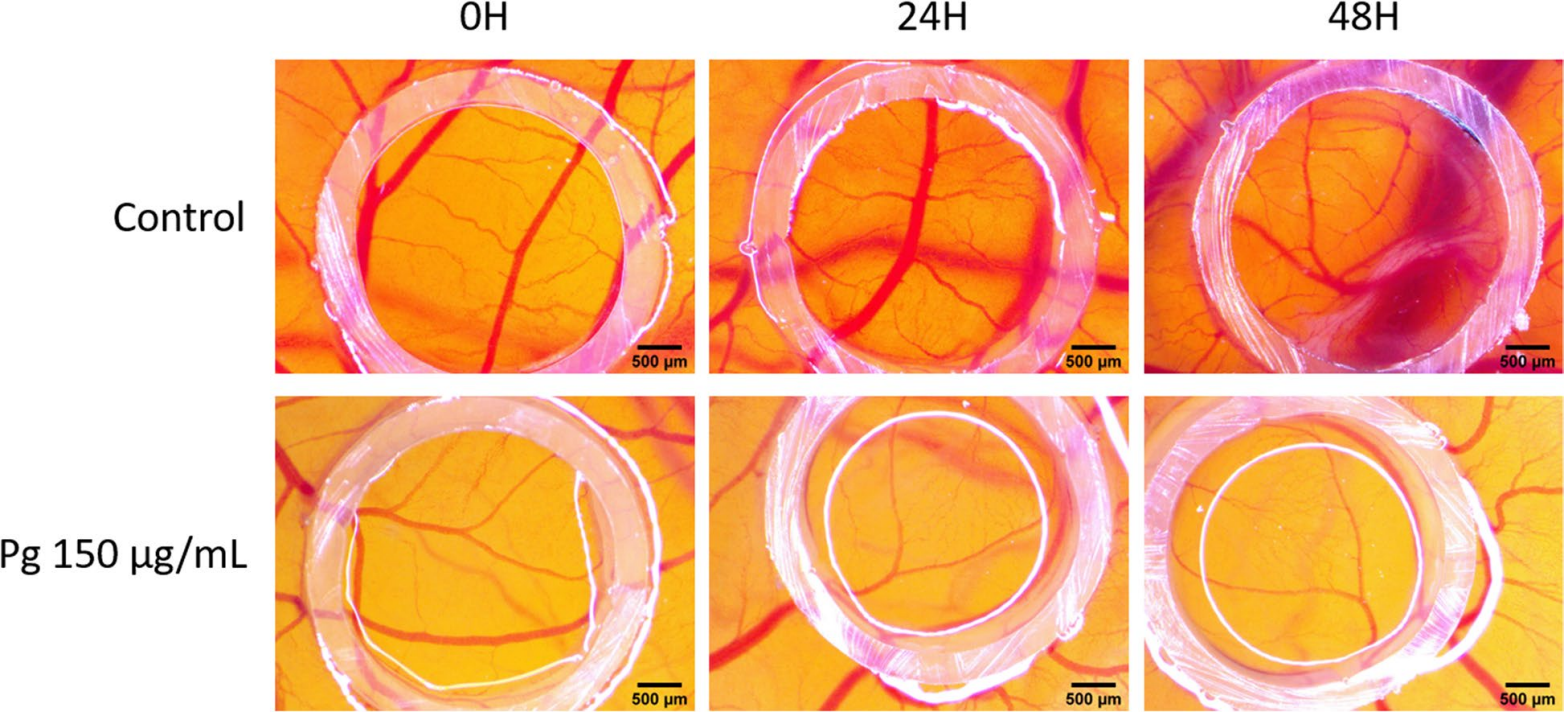
Effects of Pg extract on chorioallantoic membrane. Pg extract (150 μg/mL) and DMSO as control at 0 h, 24 h, 48 h after application; stereomicroscope images, scale bars, 500 μm

We investigated the potential implication of a Pg extract in the process of neovascularization using a largely applied in vivo method that involves an intensively irrigated extraembryonic membrane. The protocol allows the investigation of modification induced by the test samples on the normal process of angiogenesis, and moreover, by choosing as application interval between the embryonic days of development (EDD) 7–11, the compounds are evaluated on a rapidly developing capillary bed [31]. Therefore, the evaluation is highly useful as prescreening for tumor angiogenic assays. The experimental setting also allows to detect potential bioavailability and toxicity of the administered solutions upon mucosal tissues. Pg extract in the concentration of 150 μg/mL was well tolerated, no signs of toxicity upon vascular plexus were signaled in terms of coagulability. Still, some modifications upon vessel development were observed. Already after only one dose and 24 h of contact with Pg samples, areas inside the application ring on CAM showed a reduced degree of capillary interconnection (Fig. 12). Inside the application spot there are areas with a very low number of newly formed vessels, when compared to non-treated areas. Also, when evaluating the control sample, a higher degree of developing vascularization is observed inside the ring, 24 and 48 h after application.

### Immunomodulatory effects

#### Cell viability

**Fig. 13:**
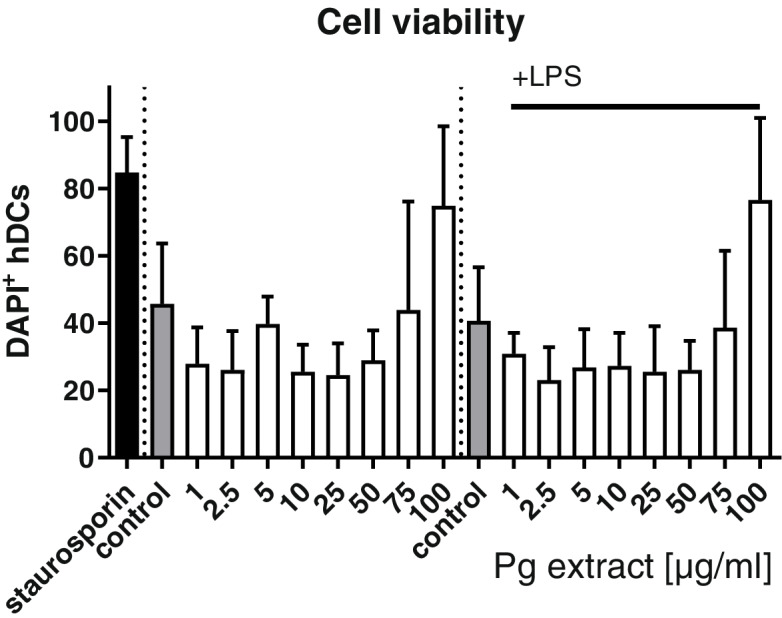
Effect of the Pg extract in different concentrations on the cell viability of naïve and inflammatory primary human dendritic cells 24 h after stimulation. Comparison among groups was made using One-way ANOVA test (*n* = 4)

**Fig. 14:**
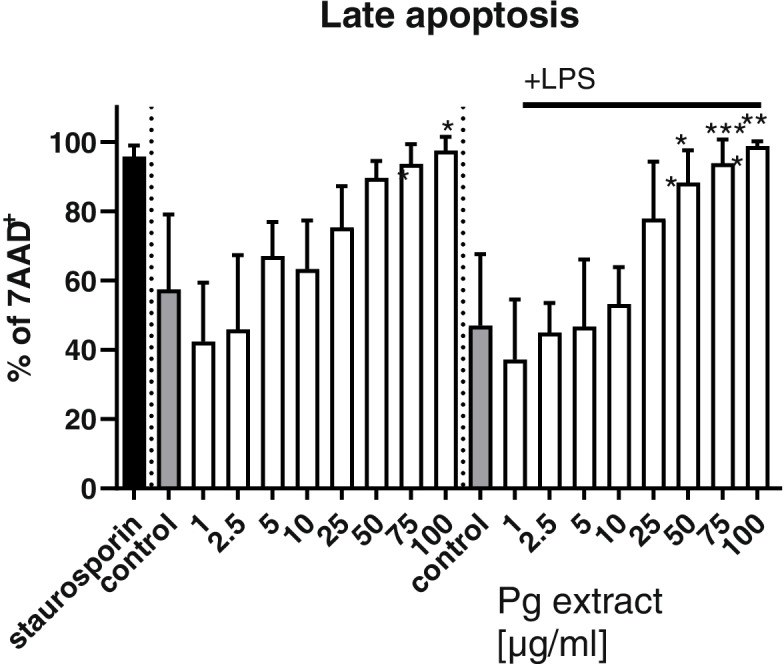
Induction of late apoptosis after stimulation with the Pg extract in different concentrations for 24 h of naïve and inflammatory primary human dendritic cells. Comparison among groups was made using One-way ANOVA test. (* *p* < 0.05, ** *p* < 0.01, *** *p* < 0.001 vs. Control)

**Fig. 15:**
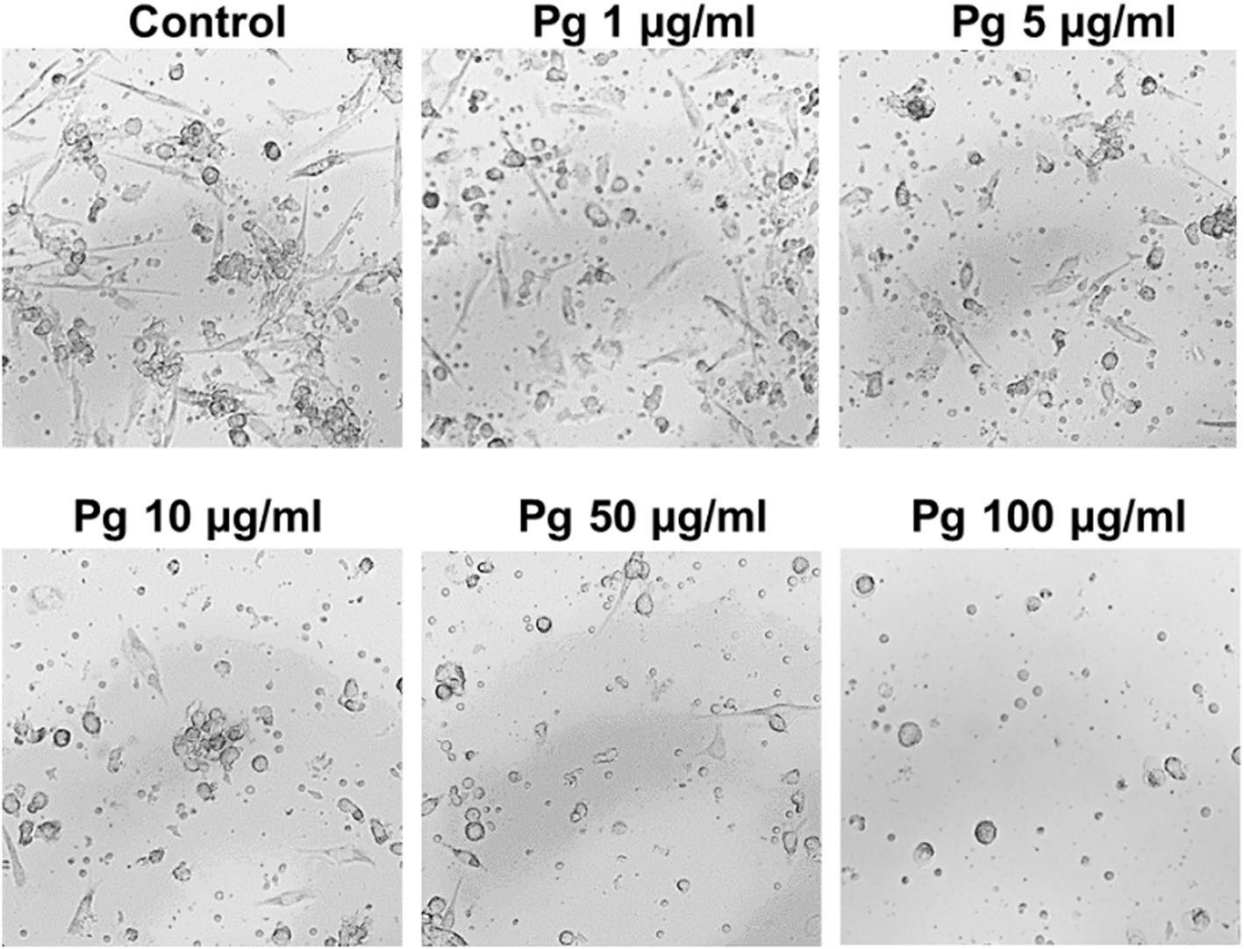
Light microscopic pictures of inflammatory human primary dendritic cells after 24 h of stimulation with the Pg-extract with indicated concentrations

To investigate the immunomodulatory effects of the Pg extract, human primary peripheral blood mononuclear cells (PBMCs) were isolated from blood, differentiated into dendritic cells (DCs), and stimulated with the Pg extract. In this set of experiments, we included also lower concentrations of the Pg extract (1–10 μg/mL), since immune cell activity and cytokine release can be very sensitive to slight stimulation. The cell viability of the dendritic cells was assessed with FACS after 24 h of stimulation with the Pg extract in different concentrations or a DMSO control, in the absence (naïve) or presence of an inflammatory stimulation with LPS (inflammatory). DAPI staining revealed no cytotoxic effect of low and moderate Pg extract concentrations on naïve and inflammatory DCs (Fig. 13). Very high concentrations (75 and 100 μg/mL) seemed to induce cell toxicity in some experiments, not reaching statistical differences. To go in more detail, cells were assessed further for late apoptosis (Fig. 14). From the data it is clear, that only cells stimulated with very low concentrations of the extract (1–10 μg) did not enter the apoptotic pathway. Especially in inflammatory DCs, concentrations above 25 μg/ml lead to the induction of apoptotic processes in the cells. Representative transmitted light microscopic images of Pg extract-stimulated human dendritic cells are presented in Fig. 15 and strengthen the impression that high Pg extract amounts are harmful to cell morphology. With increasing Pg extract concentrations, inflammatory cells start to round up and lose their spindle-like shape.

#### Cytokine expression and release

**Fig. 16:**
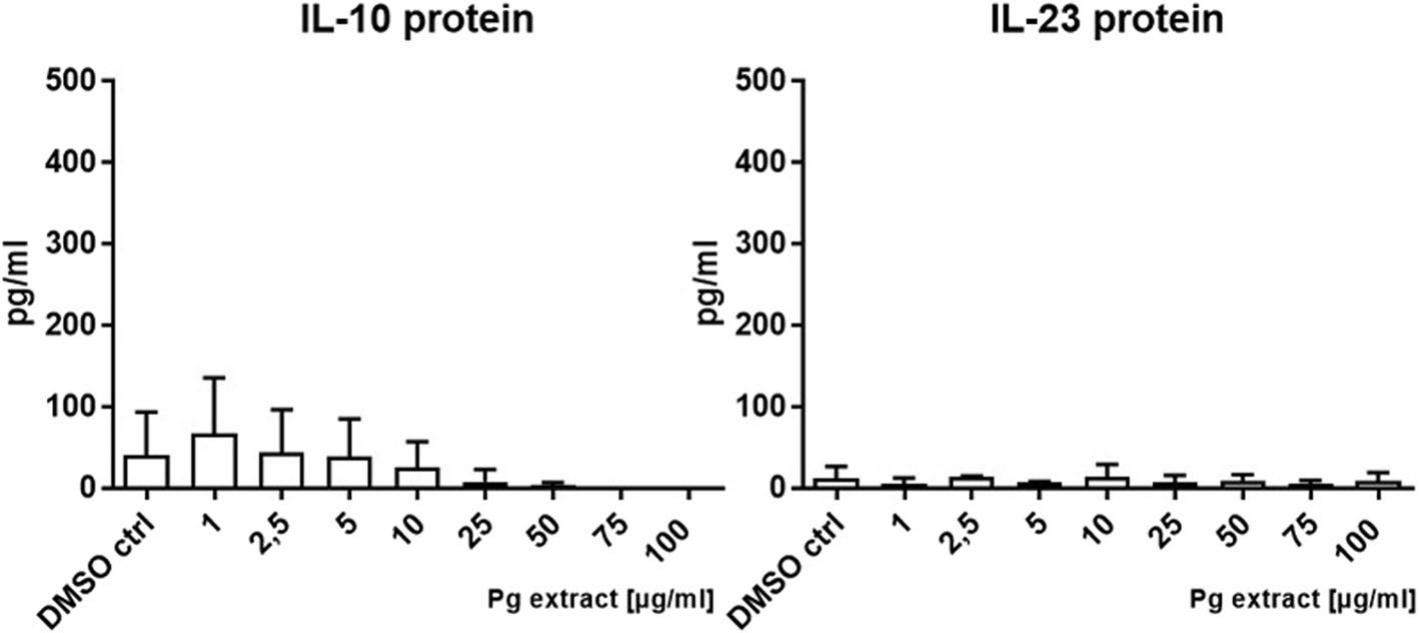
Effect of the Pg extract in different concentrations on the cytokine release (IL-10 and IL-23) of naïve primary human dendritic cells 24 h after stimulation. Comparison among groups was made using One-way ANOVA test

Cytokine secretion was analyzed in order to see if the Pg extract stimulation had functional consequences on DCs. The Pg extract was not able to activate naïve DCs (Fig. 16).

**Fig. 17:**
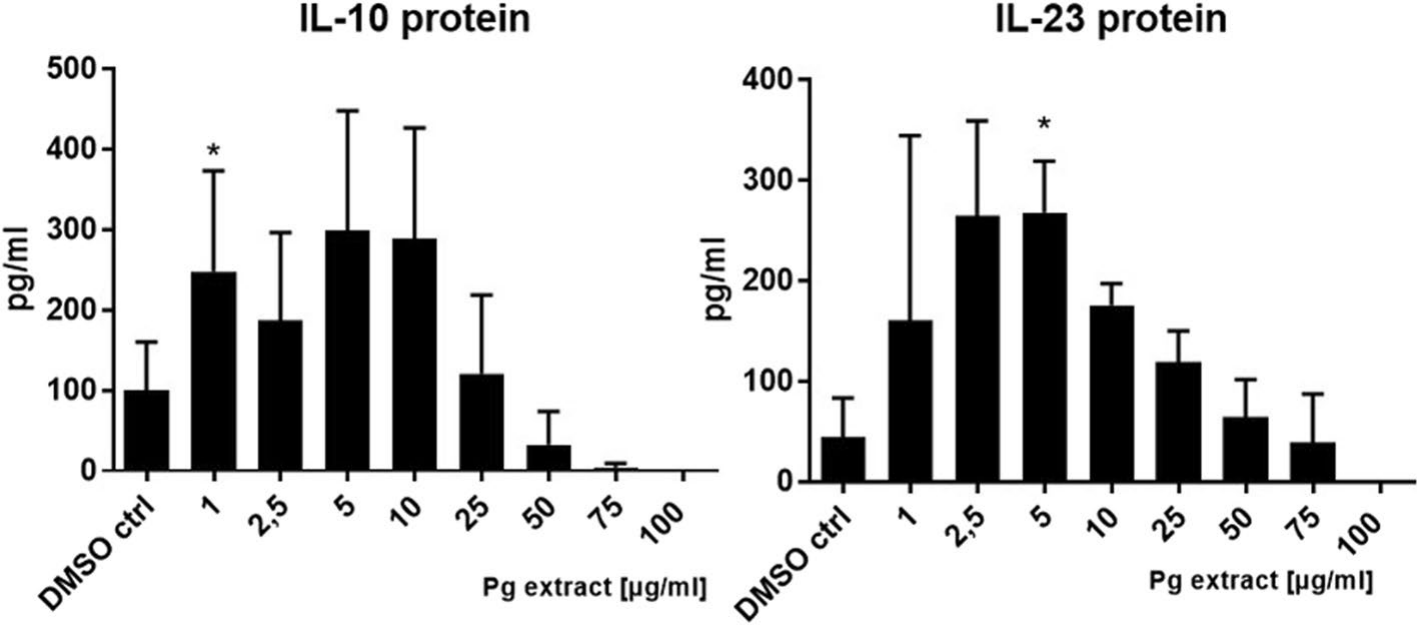
Effect of the Pg extract in different concentrations on the cytokine release (IL-10 and IL-23) of inflammatory primary human dendritic cells 24 h after stimulation. Comparison among groups was made using One-way ANOVA test. (* *p* < 0.1 vs. Control)

A significant increase in IL-10 and IL-23 secretion was achieved with low Pg extract concentrations on inflammatory DCs (Fig. 17).

**Fig. 18:**
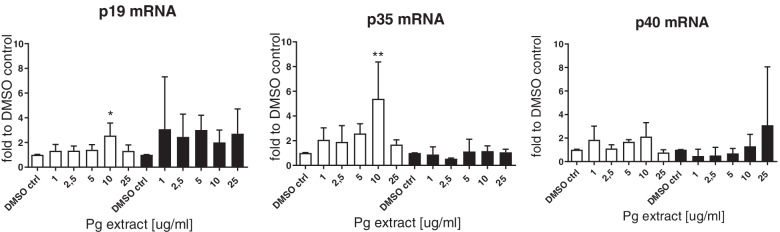
Effect of the Pg extract in different concentrations on the mRNA expression of the cytokine subunits p19, p35 and p40 of naïve and inflammatory primary human dendritic cells 24 h after stimulation. Comparison among groups was made using One-way ANOVA test. (* *p* < 0.1 vs. Control, (** *p* < 0.1 vs. Control)

Very high Pg extract concentrations (75 and 100 μg/mL) however decreased cytokine production in inflammatory DCs. On messenger Ribonucleic acid (mRNA) level we analyzed the expression of the IL-12 and IL-23 p19, p35 and p40 and found that both, p19 and p35 mRNA levels were significantly upregulated with an extract concentration of 10 μg/ml in naïve DCs (Fig. 18). On already activated DCs, the Pg extract had no effect on mRNA expression of the cytokines.

## Discussion

In the same research topic, the phytochemical composition of the ethanolic extract of Romanian poplar buds was detailed in a previous paper recently published by our research group. Briefly the extract was found to contain mainly the following phenolic compounds: dihydroxybenzoic acid, protocatechuic acid, 3-caffeoylquinic acid, 5-caffeoylquinic acid, caffeic acid, chicoric acid, apigenin-glucuronide, chrysoeriolglucuronide, tremuloidin, salicin, pinostrobin, and tremulacin [32]. In a similar approach, Greenway et al. tested the chemical composition of *Populus nigra* L. bud exudates from 7 countries (Netherlands, Russia, Belgium, United Kingdom, France, Italy and China) and reported that all screened samples contain high levels of caffeic and isoferulic acids on one hand but low levels of cinnamic and coumaric acids on the other hand. Only one sample (United Kingdom) showed an increased percentage of pinocembrin, pinobanksin, chrysin and also galangin [33]. In a comprehensive study, Jerković et al. described the phytochemical composition of volatile components from fresh and air-dried leaf-buds of *Populus nigra* L. (Croatia). They reported the presence of more than 48 phytocompounds, among which sesquiterpenes (β-eudesmol and α-eudesmol, γ-cadinene, α-elemene) and in a low percentage monoterpene, aliphatic and aromatic alcohols, carbonyl compounds have been detected [7]. The water, chloroform, methanol and hexane extract of black poplar buds (Algeria) indicate a rich source of flavonoids, polyphenols, terpenoids, polyphenols and also tannins. On the contrary, beside these compounds the author reported an absence of saponosides and anthocyanins [34]. In a recent study Ristivojević et al. reported that the ethanolic extract of poplar bud (Serbia) contains phenolic acids and their derivates (caffeic acid, p-coumaric acid), flavonols, flavanonols, flavan-3-ols, glycosides (apigenin-7-O-glucoside) and phenolic glycerides [6]. Other studies mentioned higher flavonoids (pinocembrin, pinobanksin) and phenolic acids (caffeic, cinnamic, coumaric, ferulic acid) content for the aqueous black poplar bud extract (Bulgaria) [35]. In a similar study conducted by Rubiolo et al., the black poplar bud absolute (France) exhibited a high presence of flavonoids and phenolic acids including chrysin, galangin, pinocembrin, pinostrobin and also tectochrysin [36].

As revealed in the results section the screened Pg extract shows a significant antioxidant potential. The DPPH assay is the most widely used method of determining the AOA of natural extracts, which is based on the decolorization of the DPPH solution from deep purple to pale yellow in the presence of a hydrogen donor, establishing the degree of inhibition of free radicals [17]. Results from this study are in agreement with those obtained by Mainar et al. Using the same assay (DPPH) this research group evaluates the antioxidant activity of the ethanolic extract of black poplar bud (Spain) concluding that this extract is a natural source of antioxidants [37]. Merghache et al. also pointed out the antioxidant activity of the hydroalcoholic extract of black poplar bud (Algeria). They revealed that the highest antioxidant potential was achieved at 1000 μg/mL, comparable with the ascorbic acid activity [34]. Staying in the same directions, Debbache et al. have exploited the antioxidant potential of seven extracts (ethanol, hexane, aqueous of ethyl acetate, ethyl acetate, aqueous of hexane, chloroform and aqueous of chloroform) of black poplar buds (Algeria). As revealed by their study the aqueous fraction of chloroform extract presents the highest activity and it can be used as an accessible source of natural antioxidant [38].

To the best of our knowledge, this is the first study focusing on the evaluation of Pg extract, including the thermal behavior and the FT-IR spectroscopy analysis. The results of the present study are in agreement with literature data, although most research studies are focused on the quantitative identification of active compounds from different poplar bud preparations (extraction, infusion, essential oil). The FT-IR analysis performed in the present study demonstrated the identification of the same active compounds, such as: flavonoids/flavonols (pinocembrin and pinostrobin); phenolic acids; phenolic glycosides (salicilin, populin); tannins [7, 14, 39–43]. Indeed, the FT-IR analysis is not sufficient. In order to establish to which active compound the biological effects are due, the identification and quantification with high precision it’s necessary, this being possible only through the high-performance liquid chromatography (HPLC) technique. According to the FT-IR analysis, the active compounds contained in the dried extract were phenolic acids; phenolic glycosides (salicilin, populin); flavonoids/flavonols (pinocembrin and pinostrobin); tannins. The active compounds present in Pg dried extract have different degradation temperatures. For instance, Cheng and collaborators have investigated the decomposition of five phenolic compounds in high temperature water [44]. The authors found that three phenolic acids were decomposed at 250 °C and the other two were completely degraded until 350 °C. In the study conducted by Chaaban and co-workers [45], the authors have investigated the effect of heat processing on thermal stability and antioxidant activity of six flavonoids. The complete degradation of the investigated flavonoids was in the range of 50–130 °C.

As indicated by other research group, the decomposition profile of the tannins starts at approx. 200 °C in one stage [30]. In the first stage, the maximum weight loss of the tannins degradation occurs but, in the case of condensed tannins, with a complex aromatic structure, the thermal degradation is almost complete at 600 °C [30]. Considering the exothermic process recorded on the DSC curve (547.3 °C), we can affirm that this thermal effect is associated with the degradation of aromatic compounds contained in condensed tannins from Pg dried extract, occurring in one stage. In the present study, the fact that up to 400 °C no thermal effect was recorded on the DSC curve, is due to the low content of phenolic acids and flavonoids contained in the Pg dried extract. Their presence in the extract is confirmed by the loss of mass, which is really low, in the first three stages of the analysis.

Black poplar buds possess a wide range of biological activities, among which the most important are their antioxidant, antimicrobial, anti-proliferative and anti-inflammatory properties [46]. On the other hand, similar approaches showed same biological and therapeutic activities for other natural compounds, these results increasing the interest for these researches [47–51].

In the last years, several studies have reported that poplar bud extracts present significant antimicrobial and antifungal activity. As previously mentioned, using the disk diffusion method, data presented in this study showed a remarkable antimicrobial activity against Gram-positive pathogens with important inhibition diameter for *Streptococcus pyogenes* (23 mm), *Streptococcus mutans* (26 mm) and also *Candida* species (17 mm). Among the screened strains Pg extract was active only on the Gram-positive strains, the best antibacterial effects being detected against *Streptococcus pyogenes*, *Streptococcus mutans* (both with an MIC and MBC of 0.312 mg/mL), followed by *Staphylococcus aureus* (MIC = 0.625 mg/mL) and *Candida* strains (MIC = 1.25 mg/mL). The study published by Vardar-Unlu et al. reported a similar antimicrobial activity. They tested the Pg methanolic extract against selected Gram-positive bacteria, the most sensitive being *Streptococcus pyogenes* (MIC of 0.50 mg/ml^− 1^), *Staphylococcus aureus* (MIC of 0.50 mg/ml^− 1^), *Enterococcus faecalis* (MIC of 1.00 mg/ml^− 1^) [13]. Additionally, Boumghar et al. have concluded that 100 μL of poplar bud methanolic and ethanolic extract (Algeria) elicits antimicrobial (using the disk diffusion method) and antibiofilm activity (carried out in a 96-well plate) for *Staphylococcus aureus* and *Bacillus subtilis* strains. They demonstrated that the extracts have antibacterial properties against *Staphylococcu aureus, Bacillus subtilis* and *Escherichia coli* with diameters ranging from 6.6 to 21.3 mm [52]. Debbache et al. concluded a study regarding the antimicrobial activity of black poplar buds (Algeria). Their results indicate that the organic extracts exhibited effective antibacterial activity against *Escherichia coli, Staphylococcus aureus, Pseudomonas. aeruginosa, Bacillus subtilis* and *Klebsiella pneumoniae* with inhibition zones ranging between 10 and 17 mm in comparison with Chloramphenicol and Gentamicin. On the other hand, the extracts show moderate activity against the fungal strains, *A. niger* and *F. polyferatum* with inhibition zones between 6 and 9 mm [38]. De Marco et al., reported that the ethanolic extract obtained from poplar bud present an antibacterial potential against *Pseudomonas aeruginosa* (MIC of 125 μg/mL) and also shows a strong inhibition of biofilm formation [53]. The present study based on Romanian poplar bud ethanolic extract demonstrate the in vitro antibacterial activity against Gram-positive bacteria, such as *Staphylococcus aureus* (inhibition zone diameter is 13 mm), and also *Listeria monocytogenes* (inhibition zone diameter is 15 mm) [54].

One of the main objectives of this study was to evaluate the potential use of Pg extract as a possible antiproliferative and proapoptotic agent against MCF-7 human breast cancer cell line. To the best of our knowledge this approach was never conducted before. In the present work we demonstrate that tested extracts elicit a dose-dependent decrease of tumor cell viability with an IC_50_ of 66.26 μg/mL. Moreover, data have shown that the screened extracts are selective on tumor cells. Although there is a lack of data regarding the black poplar buds antitumoral activity on different cell lines, an increased number of studies have described that *Populus nigra* L. buds are rich in phytocompounds with anticancer potential. One of these compounds is pinostrobin which was evaluated by Sukardiman et al. In this study the research group assessed the antiproliferative potential against T47D human breast cancer cell line for 10, 50, 100 μg/mL of pinostrobin with check points at 24 h, 48 h and 72 h. The results indicated that this compound has antitumor activity, moreover it increases the percentage of apoptotic cells [55]. Jaudan et al., investigated the in vitro apoptotic potential of pinostrombin on HeLa cervical cancer cell line and demonstrated a dose dependent growth inhibition potential [56]. Zheng et al., demonstrate that the pinocembrin reduces tumor volume and weight in B16F10 human melanoma cells in vivo on an experimental animal model C57BL/6 mice strain [57].

Apigenin is a natural flavone which is also a part of black poplar bud chemical composition. Cao X et al. demonstrated that apigenin (10, 20, 40, and 80 μM) induced apoptosis on T47D and MDA-MB-231 breast cancer cells in a dose and time dependent manner. Also, they reported an increased level of Bax (apoptosis regulator), decreased level of B-cell lymphoma 2 (Bcl-2) and cleaved Caspase 3 and poly ADP-ribose polymerase (PARP) molecules [58]. As discussed in the results section, we have demonstrated that on MCF-7 human breast adenocarcinoma cells the Pg extract at the highest doses (100 and 150 μg/mL) inhibits the cell migration and proliferation, furthermore it also generates changes in tumor cell morphology. Similar conclusion was drawn by Gao et al. for pinocembrin. The phytocompound (100–200 μM) inhibits in vitro the proliferation, migration, and promotes apoptosis on SKOV3 human ovarian cancer cells by down regulating the mRNA levels of N-cadherin and also the gamma-aminobutyric acid (GABAB) receptor [59].

The study published by Buahorm et al. describes the in vitro anticancer potential of cardanol, one of the components of propolis derived from bees foraging on black poplar (*Populus nigra* L.). Using MTT assay, flow cytometric analysis of Propidium iodide and Annexin-Vstained assay, they concluded that this compound induces a time and dose dependent cytotoxicity on BT474 human breast cancer cell line. They also detect that after 72 h of treating the cells with cardanol, it causes cell cycle arrest at the G1 subphase and significant numbers of cells were death by late apoptosis (27.2% ± 1.1%), compared to a lower proportion of apoptosis (4.3 ± 0.4%) and higher proportion of necrosis (35.8 ± 13.0%) induced by doxorubicin [60]. Along the same line of research Aru B et al. have published that propolis originated from poplar buds exerted a significant cytotoxic activity against MCF-7 breast cancer and A549 lung cancer cell lines with an IC_50_ among 58.6–90.7 μg/mL, compared with Paclitaxel. Treatment with the ethanolic extract of poplar type propolis significantly decreased viability while promoting apoptosis in both cell lines. Regarding cell cycle arrest in cancer cells, MCF-7 has the highest sub-G0 cell population rate [61]. This phenomenon was also observed in our study. Our data shows that 150 μg/mL Pg extract induces apoptosis in a significant manner. The highest number of events was executed for the process of early apoptosis. Chicoric acid, another phenolic compound of *Populus nigra* L. bud obtained in this study by LC-MS analysis was reported as a chemotherapeutic agent. Tsai et al. reported that this compound (50, 150 and 200 mg/mL) induces apoptosis in HCT-116 colon cancer cell line which was described by DNA fragmentation, activation of Caspase-9 and cleavage of PARP [62]. Recently, Sun et al. has indicated that autophagy plays an important role in the progression of different diseases, especially tumors. They elucidated that chicoric acid (5, 10, 20, 40, 80 and 100 μM) significantly reduced the SGC7901 and MGC803 human gastric cancer cell viability, prevented tumor cell growth and also induced autophagy through the activation of AMPK [63].

Excessive activation of angiogenesis is one of the key features explaining tumor growth, highly metastatic rates and the poor prognosis of breast cancer. Natural compounds are an important source of potential multitargeted effects capable of controlling breast cancer progression [64]. Our evaluation indicated that poplar bud extracts in the concentration of 150 μg/mL was well tolerated, no signs of toxicity upon mucosal tissues were noted, while inducing 24 h after inoculation, a reduced number of newly formed vessels. Thus, indicating a potential antiangiogenic effect upon the rapid growing vascular net of the chorioallantoic membrane.

Little data is available on the effect of poplar leaf or bud extracts as angiogenic modulators. One study showed that an aqueous extract of *Populus nigra* L. leaves induced an activation of cutaneous angiogenesis reaction in mice induced by human mononuclear leukocytes [65]. Others have shown that extracts of poplar buds induce a vascular relaxation not related to endothelial cell activity [66]. More data regarding the angiogenesis effects is available for various poplar propolis, the most widely available type of propolis from temperate climates. The chemical profile of poplar propolis is mainly formed by flavonoids, phenylpropanoids, terpenoids, stilbenes, lignans, coumarins, with lower concentrations of phenolic acids [67]. Some types of Brazilian green propolis type, rich in artepillin C (3,5-diprenyl-4-hydroxycinnamic acid) were studied and antiangiogenic effects were recorded. Other studies on different red and green Brazilian propolis showed that polyphenols were responsible for down regulating genes involved in angiogenic signaling cascade, such as angiopoietin I, angiopoietin II, vascular endothelial growth factor (VEGF), fibroblast growth factor, metalloproteinases 2 and 9, platelet-derived growth factor, thus reducing angiogenesis and the inflammatory process [68]. Mechanistic data were reported by Kunimasa et al., showing the inference of some non-poplar Brazilian propolis extracts in inhibiting the survival signal Extracellular Signal-Regulated Kinases 1 and 2 (ERK1/2), thus inducing apoptosis that is critically involved in angiogenesis suppression [69]. Another pathway influenced by propolis as indicated by some wound healing studies is the effect on mast cells as known promoters of the angiogenesis process. Propolis decreased mast cell count and the secretion of pro-inflammatory and pro-angiogenic cytokines was therefore reduced [67]. Another study evaluated the effect Portuguese propolis on breast and prostate cancer cells and the antiproliferative effect of paclitaxel was enhanced in vitro, while reducing the neovascularization from preexisting vessels in a CAM assay, possibly due to the derivatives of caffeic acid [70].

Data on the immunomodulatory effect of the Pg extract revealed an effect of low concentrations of the extract on cytokine release by human primary dendritic cells. P35 and p19 are subunits of the IL-12 and IL-23 cytokines. P35 is forming together with p40 the cytokine IL-12, while p19 and p40 form IL-23. Both cytokines are believed to direct tumor-regulating immune responses. While IL-12 is believed to achieve an anti-tumor Th1-driven inflammatory response, IL-23 is believed to favor a Th17-driven immune response [71]. Since both cytokine subunits are significantly upregulated, it is not clear which immune response will be favored by the Pg extract. From the protein data in this study, it was clear, that IL-23 release was slightly upregulated by the extract. IL-12 measurement failed in our experiments, since levels were under the limit of quantification. On the other hand, IL-10 levels were slightly increased as well. IL-10 is known to have a suppressive effect on the immune system being an important regulator of immune tolerance [72]. It thus needs to be elucidated in which direction the Pg extract will drive immunomodulation or if a general activation of the immune response is achieved by the phytocompound.

The increase in cell toxicity and the development of apoptotic processes in the presence of higher Pg extract concentrations is indicated by the constantly decreased release of cytokines with rising amounts of the extract. Apoptosis staining confirmed these results.

## Conclusion

To the best of our knowledge this is the first comprehensive study that includes data regarding both the antioxidant activity, along with the biological activity of poplar bud extract obtained from the western part of Romania on breast adenocarcinoma and dendritic cells. The extract has inhibitory activity against Gram-positive bacteria, *Streptococcus pyogenes* and *Streptococcus mutans*, followed by *Staphylococcus aureus*. The comprehensive assessment of the effect of Pg extract against MCF-7 human breast cancer cell line indicates that under experimental conditions, the extract may exert an anticancer effect through the inhibition of cell proliferation and migration and the induction of early apoptotic events. Moreover, the extract showed antiangiogenic potential on the chorioallantoic membrane. In low concentrations, the Pg extract has also an immunomodulatory potential on human primary dendritic cells.

## Data Availability

All data used to support the findings of this study are included within the article.

## Abbreviations

AOA: Antioxidant activity
ATCC: American Type Culture Collection
BAX: Apoptosis regulator
BCL-2: B-cell lymphoma 2
BF: Bright field
CAM: Chorioallantoic membrane
CFU: Colony forming units
CLSI: Clinical Laboratory and Standard Institute
DAPI: 4′,6-diamidino-2- phenylindole
DCs: Dendritic cells
DMSO: Dimethyl sulfoxide
DNA: Deoxyribonucleic acid
DPPH: 2,2-diphenyl-1-picrylhydrazyl
EDD: Embryonic day of development
ERK1/2: Extracellular Signal-Regulated Kinases 1 and 2
EUCAST: European Committee on Antimicrobial Susceptibility Testing
FBS: Fetal bovine serum
FT-IR: Fourier-transform infrared spectroscopy
GABAB: Gamma-aminobutyric acid
GAPDH: Glyceraldehyde 3-phosphate dehydrogenase
HGF-1: Insulin-like growth factor 1
HPLC: High-performance liquid chromatography
IC_50_: Half-maximal inhibitory concentration
LC: Liquid chromatography
LDH: Lactate Dehydrogenase
IL: Interleukin
LPS: Lipopolysaccharide
MIC: Minimum inhibitory concentration
MBC: Minimum bactericidal concentration
MH: Mueller Hinton
mRNA: messenger Ribonucleic acid
MS: Mass spectrometry
MTT: 3-(4,5-dimethylthiazol-2-yl)-2,5-diphenyltetrazolium bromide
NF-κB: Nuclear factor kappa B
PARP: Poly ADP-ribose polymerase
PBMCs: Primary peripheral blood mononuclear cells
PBS: Phosphate Buffer Saline
Pg: *Populus nigra* L. bud extract
PI: Propidium iodide
Rt: Retention time
TG-DSC: Thermogravimetric Analysis-Differential Scanning Calorimetry
TNF-α: Tumor necrosis factor alpha
UV-VIS: Ultraviolet –visible
VEGF: Vascular endothelial growth factor
